# Engineered cell-cell communication via DNA messaging

**DOI:** 10.1186/1754-1611-6-16

**Published:** 2012-09-07

**Authors:** Monica E Ortiz, Drew Endy

**Affiliations:** 1 Bioengineering Department, Stanford University, Y2E2 Room 269B, 473 Via Ortega, Stanford, CA, 94305-4201, USA

**Keywords:** Synthetic biology, Amorphous computing, Cell-cell signaling, Programmed pattern formation, Communication theory

## Abstract

**Background:**

Evolution has selected for organisms that benefit from genetically encoded cell-cell communication. Engineers have begun to repurpose elements of natural communication systems to realize programmed pattern formation and coordinate other population-level behaviors. However, existing engineered systems rely on system-specific small molecules to send molecular messages among cells. Thus, the information transmission capacity of current engineered biological communication systems is physically limited by specific biomolecules that are capable of sending only a single message, typically “regulate transcription.”

**Results:**

We have engineered a cell-cell communication platform using bacteriophage M13 gene products to autonomously package and deliver heterologous DNA messages of varying lengths and encoded functions. We demonstrate the decoupling of messages from a common communication channel via the autonomous transmission of various arbitrary genetic messages. Further, we increase the range of engineered DNA messaging across semisolid media by linking message transmission or receipt to active cellular chemotaxis.

**Conclusions:**

We demonstrate decoupling of a communication channel from message transmission within engineered biological systems via the autonomous targeted transduction of user-specified heterologous DNA messages. We also demonstrate that bacteriophage M13 particle production and message transduction occurs among chemotactic bacteria. We use chemotaxis to improve the range of DNA messaging, increasing both transmission distance and communication bit rates relative to existing small molecule-based communication systems. We postulate that integration of different engineered cell-cell communication platforms will allow for more complex spatial programming of dynamic cellular consortia.

## Background

The importance of cell-cell signaling throughout nature [1] suggests that better tools for engineering cell-cell communication will advance biotechnology. For context, other engineering disciplines have recognized the importance of understanding and programming systems across both space and time, and have even begun to map such work to biological substrates. As one example, Amorphous Computing (AC) was developed to help program system-wide patterns and other emergent behaviors within networks of independent elements [2]. Drawing inspiration from biology, AC-based systems are comprised of identical but autonomous agents that make use of local information to differentiate and act across space and time. The resulting space-time programming frameworks enable both the analysis and forward engineering of complex systems capable of programmed pattern formation and restoration (i.e., “healing”) in the presence of noise or damage [3-5]. However, much improved cell-cell communication platforms are needed for scientists and engineers to practically benefit from and advance such research.

**Figure 1:**
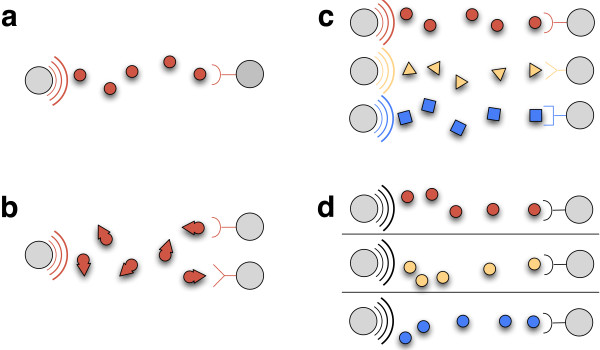
**Existing cell-cell communication systems are limited by message-channel coupling.** Four alternate schemes for engineering cell-cell communication. Gray circles represent cells. Colored arcs on left cells represent transmitters with different colors representing distinct transmitters. Stick-drawings on right cells depict receivers. Icons passing between cells represent information channels with colors representing distinct messages. **(a)** Single channel coupled to specific message (e.g., AHL-based systems), **(b)** Single channel coupled to specific message but with multiple receivers (e.g., AHL-based systems in which signaling molecule is recognized by distinct cognate response factors), **(c)** Multiple insulated channels each coupled to a specific message (e.g., a set of AHL-based systems with no crosstalk between signaling molecules and cognate response factors), **(d)** Single channel decoupled from any one message (e.g., M13-based systems, this work).

Autonomous engineered cell-cell communication was first demonstrated via the directed transmission of acyl-homoserine-lactones (AHLs) (Figure 1a) [6]. AHLs are freely-diffusing small molecules found in natural quorum sensing systems of various bacteria [7]. Subsequent pioneering examples have included Weiss et al.’s use of AHL concentration gradients to establish two-dimensional patterns [8], Tabor et al.’s engineering of an edge detector by integrating AHL-based signals to produce patterns in response to external stimuli [9], You et al.’s creation of synthetic predator–prey ecologies [10,11], and Danino et al.’s synchronization of genetic oscillators within microbial populations [12]. Most recently, Tasmir et al. demonstrated how cell-cell communication can be combined with serial plating of distinct cell types in order to approximate Boolean logic [13].

**Figure 2:**
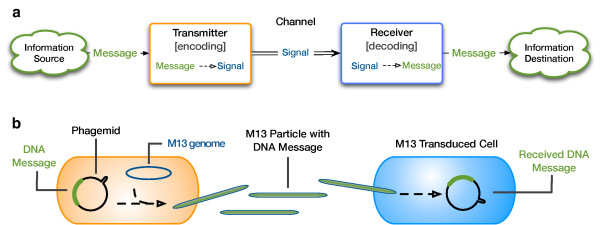
**Biological communication systems can be represented formally. (a)** Schematic diagram of a general communication system, adapted from Shannon (19). **(b)** Representation of a bacteriophage M13-based cell-cell communication system within Shannon’s framing.

Most modern communication systems are based on the theoretical work of Shannon [14]. We thus mapped existing examples of engineered cell-cell communication into Shannon’s framework in order to identify possible areas for fundamental improvement (Figure 2a). For example, of the engineered AHL-based systems noted above, an AHL signaling molecule is either directly added to cells or transmitted from sender cells to receiver cells. The “transmitter” in these systems is the source of signaling molecules, the researcher or encoded AHL synthase. The communicated “message” is, directly or indirectly, to transcribe genes under the control of a cognate signaling pathway or promoter. The message itself is encoded via the AHL concentration and the information “channel” is diffusion of AHL molecules. The “receiver” is the cognate receptor or transcription factor within receiver cells, which directly or indirectly initiate transcription in the presence of AHL.

Two limitations become immediately apparent in considering existing engineered AHL-based systems within Shannon’s framing. First, such systems can transmit only a single message for a given information channel (Figure 1a). This “single message-single channel” limitation is due to the required molecular interaction between a given AHL and its cognate receptor or transcription factor. Attempting to encode a distinct message by changing the chemical structure of an AHL signaling molecule can adversely affect its interaction with its cognate receiver and disrupt the communication channel itself. In other words, the first limitation encountered in AHL-based systems is that the message and information channel are coupled such that the message is the molecule. Second, AHL-encoded messages are known to result in, directly or indirectly, regulation of transcription. Thus, AHL-based receivers can realize the receipt of just a single message type, “regulate transcription.” Stated differently, the second limitation of AHL-based systems is their inability to transmit more than a single type of message.

One method for overcoming message-channel coupling is to increase the number of available independent channels (Figure 1c). However, members of the LuxR transcription factor family often respond to multiple AHL signaling molecules, leading to non-specific interactions and interference (Figure 1b) [15,16]. To explore and address molecular interference, researchers have employed directed evolution approaches to alter interactions among LuxR transcription factors and competing AHL molecules [17-19]. For example, the specificity of a LuxR variant for a specific AHL molecule was altered to allow recognition of straight-chained AHLs and to lack recognition of AHLs with a 3-oxo group. Unfortunately, so-evolved LuxR variants were not fully insensitive to AHL molecules with 3-oxo groups [18]. Moreover, while Balagaddé et al. was able to implement two independent AHL-based communication channels, receiver-specific genetic architectures were required as both AHL-responsive transcription factors recognized the same core promoter sequence [11,19]. Practically, only two AHL cell-cell communication channels, based on the LuxR and LasR transcription factors, have now been proven for independent use within any given engineered system.

Another approach for overcoming message-channel coupling would be to establish an information channel that is capable of transmitting arbitrary messages encoded via a common format. We immediately noted that many natural organisms can directly or indirectly exchange or uptake nucleic acids [20,21], a class of biomolecules whose underlying chemistry has itself been selected to support the encoding of information defining any genetic sequence [22,23]. We then specifically chose to focus on virus-mediated exchange of genetic material, as opposed to bacterial conjugation, since virus particles are typically physically released from infected cells whereas conjugation would require that sender and receiver cells interact directly.

Many viruses are capable of packaging and transmitting non-viral genetic material [24,25]. For example, “transvestite” bacteriophage lambda particles can package and transduce phage T7 genomes or other genetic material [26,27]. However, phage lambda and many viruses actively destroy the host cell in releasing virus particles. We thus instead considered viral systems that secrete progeny without destroying the infected host cell.

Bacteriophage M13 (M13) is a filamentous phage whose progeny particles are secreted from infected cells [28]. M13 is able to package single stranded DNA (ssDNA) of various lengths, a property that has allowed for the “hard-coding” of derivative M13 genomes that express various heterologous genes, including fusion proteins and transcription factors [29-31]. M13 is also able to package independent heterologous DNA sequences that contain the M13 packaging sequence. For example, “phagemids,” plasmids encoding an phage DNA packaging sequence, have been used to generate M13 and other phage particles containing heterologous DNA that can then be applied to direct allelic replacement within *E. coli*[32-37]. However, such uses of phage particles to modify or exchange DNA typically require the physical purification or concentration of phage particles from infected cultures prior to their manual reapplication to transduce independent cultures.

Here, we repurpose the natural properties of M13 to engineer fully autonomous *in situ* cell-cell communication among bacterial cells. We show that an M13-based system can send DNA messages via a single information channel. Specifically, sender cells transmit an arbitrary DNA message that is encoded by a heterologous “messaging phagemid” and then packaged within M13 gene products. Receiver cells are transduced by so-produced M13 “message particles” containing user-defined DNA messages. We demonstrate transmission of various distinct DNA messages of varying lengths and encoded biochemical functions, including message-specific activation of genetic functions encoded within receiver cells, and long range transmission of DNA messages via active chemotaxis. Taken together, we have engineered a cell-cell communication platform that supports message-channel decoupling via phage particle-mediated transduction (Figure 1d).

## Results

We started by specifying three criteria for improving engineered cell-cell communication systems. First, a system should be “decoupled,” which we define as the ability to transmit distinct messages via a reusable channel, such that the same system can be used in diverse applications without requiring changes to the system itself. Second, a system should be “flexible,” which we define here as the ability to accommodate messages having different encoded lengths and biochemical functions, again to more readily enable applications. Finally, message transmission within the overall system should be “specific” such that certain cells can be made susceptible to message readout while others are not, so as to enable targeted cell-cell communication within mixed cultures.

We worked to meet our three criteria using M13. As noted above, we chose M13 because it can specifically package non-M13 genetic material and release so-produced particles without killing the host cell. In addition, binding of and infection by M13 particles requires cells to produce a pilus as encoded from the F-plasmid [38]. The resulting F^+^ strains are thus susceptible to transduction while F^-^ strains are not.

### Message transmission requires M13 packaging, particles, and F^+^ receiver cells

We first confirmed that message transmission occurs only if sender and receiver cells express M13 and F-plasmid gene products, respectively, and DNA molecules encode the M13 packaging sequence (Figure 2b). Stated differently, we considered if several imaginable modes of non-specific or alternate DNA exchange among cells might corrupt or bias M13-directed messaging. For such experiments we typically grew independent, well-mixed liquid cultures of sender and receiver cells to OD_600_ ≈ 0.7, combined equal numbers of sender and receiver cells to create a fresh co-culture, regrew a 1:100 dilution of the mixed population for five hours in the absence of antibiotic selection, and then regrew 1:1000 dilution split co-cultures with antibiotic selection for the messaging phagemid, receivers cells, or both (Methods).

**Figure 3:**
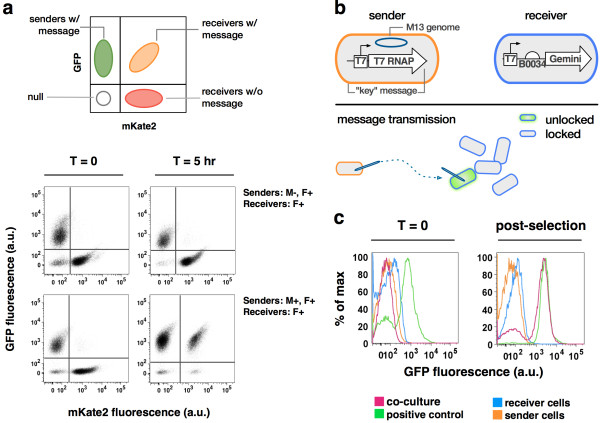
**Targeted and autonomous communication of arbitrary DNA messages via a reusable cell-cell communication channel. (a)** Bacteriophage M13-based cell-cell communication was used to send a DNA message, “GFP and ampicillin resistance”, to receiver cells constitutively expressing mKate2. Schematic shows expected locations of cell populations after flow cytometry analysis. Data show fluorescence of GFP and mKate2 of co-cultures at the start of an experiment (t = 0) and after co-culture without antibiotic selection (t = 5 hr). Data are shown for experiments using cells with varying attributes, listed to the right of each pair of plots. **(b)** Schematic showing sender and receiver genotypes and expected message transmission of a lock-and-key system. Sender cells contain and transmit “T7 RNA polymerase autogene.” Receivers contain a green fluorescent protein-lacZ alpha fragment fusion (“Gemini”) under the control of a T7 promoter. Expression of Gemini is unlocked in Receivers given message transmission (34). **(c)** Communication of a lock-and-key message demonstrating decoupling and also the capacity for specificity of decoding received messages. Percentage of maximum events as a function of GFP fluorescence are shown for sender cells (XL1-Blue, Litmus-T7 RNA polymerase), receiver cells (XL1-Blue, pSB4C5-T7promoter-Gemini), co-cultures of receiver and sender cells, and a positive control (XL1-Blue, co-transformed with Litmus-T7 RNA polymerase and pSB4C5-T7promoter-Gemini). The curves for co-culture fluorescence are pooled data from three separate co-culture flasks. Data are shown for the start of the experiment (t = 0) and after selection with antibiotics (post-selection). Additional transmitted messages (Additional file 1: Figure S6).

To test if message transmission could occur in the absence of M13, we engineered F^+^ sender cells containing a messaging phagemid expressing green fluorescent protein (GFP) and ampicillin resistance, and F^+^ receiver cells expressing a red fluorescent protein (mKate2) and a chloramphenicol resistance marker (Methods). We co-cultured an equal number of sender and receiver cells in the absence of antibiotic selection. We assayed single cell fluorescence from chemically fixed samples taken at the start of the co-culture and following five hours of growth (Methods). We found no cells expressing both GFP and mKate2, suggesting that the messaging phagemid present in the sender cells was not transmitted to any receiver cells (Figure 3a, top row). We then regrew splits of the co-culture in the presence of ampicillin, chloramphenicol, or both antibiotics. No cells grew in the combined presence of ampicillin plus chloramphenicol (Additional file 1: Figures S3, S4).

We then transformed M13K07, a “helper phage” plasmid that encodes all M13 gene products along with a weak M13 packaging sequence, to our sender cells (Methods). We repeated the experiments as detailed above and found, following five hours of co-culture in the absence of antibiotic selection, that 92.4% of receiver cells (mKate2) also expressed GFP (Figure 3a, bottom row). We found that these cells were also able to grow in the combined presence of ampicillin plus chloramphenicol (Additional file 1: Figures S3, S4), suggesting that the messaging phagemid present in the sender cells was transmitted to receiver cells via M13 message particles. We note that the M13K07 helper phage also results in the production of a small fraction of secondary “M13K07” messages that convert receiver cells to potential rebroadcasting cells (below).

To cross-validate the above observations we used F^-^ sender and receiver strains and repeated our messaging experiments in the presence of M13K07. We found no cells expressing both GFP and mKate2 (Additional file 1: Figure S2a). We also did not observe cell growth in the combined presence of ampicillin plus chloramphenicol (Additional file 1: Figures S3, S4). To confirm that M13 message particles were still being produced, we prepared a 0.2 micron filtrate from the supernatant of the initial co-culture and manually transduced separate F^+^ and F^-^ receiver cell cultures (Additional file 1: Figure S2). We found that so-treated F^+^ receiver cells gained ampicillin resistance while F^-^ receiver cells did not (Additional file 1: Figure S2b).

To confirm that the M13 DNA packaging sequence is required to package DNA into M13 particles we obtained a modified helper phage (HPdO) in which the packaging sequence is deleted [39]. HPdO sender cells should not produce any “HPdO” messages and therefore not convert any receiver cells to rebroadcasting cells. Both M13K07 and HPdO express a kanamycin resistance marker. We thus repeated our experiments using F^-^ sender and receiver cells with HPdO and found no transmission of the messaging phagemid (Additional file 1: Figure S2a). We prepared a 0.2 micron filtrate from the co-culture and manually transduced independent F^+^ and F^-^ receiver cell cultures. Like with M13K07, HPdO supernatants produced ampicillin plus chloramphenicol resistant cells only with F^+^ cultures, indicating successful messaging with either helper phage. However, only M13K07 supernatants produced kanamycin plus chloramphenicol resistant receivers, indicating that no M13 particles containing HPdO DNA were produced (Additional file 1: Figure S2). Stated differently, we did not observe any non-specific packaging of marker-encoding DNA into transducing M13 particles.

### Message channel decoupling

To demonstrate that M13-based messaging can be used to transmit arbitrary genetic sequences we engineered a new messaging phagemid encoding a weak T7 RNA polymerase autogene [40] and ampicillin resistance, and receiver cells that regulate expression of a chimeric beta-galactosidase: GFP reporter [41] via a consensus T7 promoter (Methods). We termed this type of messaging “lock-and-key” since expression of T7 RNA polymerase from the sender cell message is required to activate the receiver cell reporter (Figure 3b). We co-cultured sender and receiver cells for five hours and then added antibiotics to select those cells that received the message. We used flow cytometry to assay GFP fluorescence in the co-culture, in independent sender and receiver cultures, and in a positive control in which we directly co-transformed receiver cells with the messaging phagemid. Only the co-culture of sender and receiver cells obtained GFP fluorescence levels equal to the positive control (Figure 3c).

### Longer range messaging via chemotaxis

We then demonstrated that the range of M13-based DNA messaging can be increased using bacterial chemotaxis. For context, we confirmed by both experiment and theory that passive diffusion of M13 particles results in a detectable average run length of only ~2 mm after 24 hours on a semisolid low concentration agar plate (Additional file 1: Calculations & Figure S5). Moreover, we were initially unsure if M13 particle production, secretion, and transduction could be coupled to chemotaxis given imaginable issues arising from the combined metabolic burden of both systems, interference of partially secreted M13 filaments on flagella rotation, chemotactic shear forces destroying M13 particles during their secretion, or hindered transduction of receivers on swim plates in which chemotactic gradients are self-established via cell replication and maintenance.

The *E. coli* strain (RP437) used in our well-mixed liquid culture communication assays (Figure 3a) is chemotactic [42]. We thus obtained a non-motile strain (RP5838) for use as a control [42]. We reused the GFP plus ampicillin messaging phagemid for sender cells and the mKate2 plus chloramphenicol marker plasmid for receiver cells (Methods). In a typical chemotaxis experiment we first spotted equal amounts of sender and receiver cells on opposite sides of a 10 cm swim plate (Methods). We allowed cells to grow and move for 48 hours at 37C. We then assayed bright field plus green and red fluorescence using a tabletop imaging system (Methods). Lastly, we removed nine equally-spaced agar plugs spanning the region between the initial sender and receiver cells in order to directly observe cell phenotypes via microscopy or indirectly via antibiotic resistance in liquid culture (Methods).

**Figure 4:**
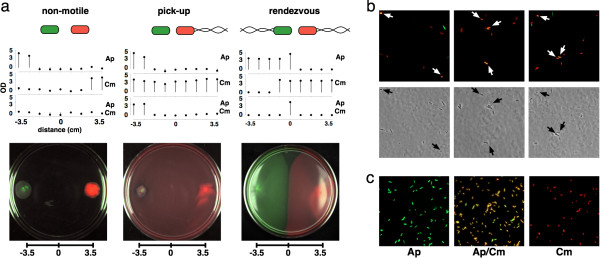
**Active chemotaxis enables DNA messaging across centimeter lengths. (a)** Bacterial chemotaxis using swim plates allows for interaction and messaging among sender and receiver cells. Three cases are presented: “non-motile” using non-motile sender and receiver cells; “pick-up” using non-motile sender cells but motile receiver cells and; and, “rendezvous” using motile sender and receiver cells. Stem plots give the measured less background absorbance (solid circles with stems) for well-mixed liquid cultures inoculated from plugs sampled in a equal spaced pattern across swim plates and cultivated with selection for sender cells (ampicillin), the messaging phagemid (chloramphenicol), or message transmission (both antibiotics). Distance from plate centers as noted; x-axes are consistent across each stem plot and cognate merged fluorescent / bright field plate photograph. **(b)** Active transmission of a DNA message across a 7 cm gap. Three representative photographs (upper row) merging RFP and GFP fluorescence from cells obtained directly from the merge region of a “rendezvous” swim plate; corresponding bright field images (lower row). Arrows indicate yellow cells expressing both reporters. **(c)** Merged multi-channel fluorescent micrographs of cells grown in replicate well-mixed liquid cultures inoculated with one sample taken from the merge zone of the rendezvous swim plate and cultivated under antibiotic selections, as noted.

We first spotted non-motile sender and receiver cells on opposite sides of swim plates (Figure 4a, left). As expected, we did not observe significant cell motility. GFP fluorescence encoded by the messaging phagemid was detected only with the sender cells. RFP fluorescence marking the receiver cells was found only on the opposite side of the plate. Liquid cultures inoculated from samples taken across the plate grew only in the presence of ampicillin (messaging phagemid within senders) or chloramphenicol (receivers), but not both antibiotics.

We then spotted non-motile senders with motile receivers (“pick-up” messaging), and motile senders with motile receivers (“rendezvous” messaging). We observed receiver cell motility and plate-wide RFP levels and chloramphenicol resistance during pick-up messaging (Figure 4a, middle). We were able only to isolate cells resistant to ampicillin plus chloramphenicol from samples taken from where both sender and receiver cell populations merge. We observed sender and receiver motility and corresponding half-plate RFP and GFP levels plus antibiotic resistances during rendezvous messaging (Figure 4a, right). We again isolated cells resistant to ampicillin plus chloramphenicol only from samples taken from where both sender and receiver cell populations merged.

We also directly imaged single cells taken from the merge zone on the “rendezvous” plate for both GFP and RFP fluorescence prior to regrowth or selection for antibiotic resistance. We observed a mixed population of green (24.4 ± 5.3%), red (48.5 ± 5.3%), and yellow (27.1 ± 5.3%) cells (Figure 4b). When we cultured this mixed population in the presence of ampicillin, chloramphenicol, or both antibiotics, we recovered clonal sub-populations specific to the expected sender, receiver, and receiver plus message phenotypes (Figure 4c).

## Discussion

We observed that over 90% of individual receiver cells were transduced via M13-mediated messaging in a well-mixed liquid culture, as evidenced by message readout within receiver cells prior to selection for message receipt (Figure 3a). We also observed that over 33% of receiver cells were transduced via M13-mediated messaging within a semisolid medium across a ~7 centimeter gap bridged by chemotaxis of sender and receiver cells, again as observed by message readout within receivers prior to selection for message receipt (Figure 4a-b). In both cases 100% message receipt could be selected for using message-specific markers (Figure 4c, Additional file 1: Figures S3, S4). We expect that lower message receipt rates in semisolid media are due to a combination of factors, including the requirement for passive diffusion of M13 messaging particles within the agar microenvironment and, in our experiments here, chemotaxis driven by nutrient depletion due to cell growth, resulting in resource-limited cells within the messaging zone and thus a presumptive limited time window for messaging [43,44]. We note that a greater than 33% messaging rate compares favorably to pioneering work with AHL wherein limited fractions of single cells responded across a few millimeter gap [45], and expect that long range M13 messaging rates can be increased (below).

M13 phage particles are ~6 nm diameter rods whose length is defined by the packaged DNA molecule. For example, the wild-type M13 genome of 6,407 base pairs (bp) produces a ~930 nm rod; the longest reported DNA fragment packaged within a M13 particle, ~40,000 bp [46], would be expected to produce a ~5.8 micron rod. For comparison, the effective radius of an AHL molecule is ~0.5 nm. Thus, when the wild-type M13 particle is modeled as a cylindrical rod, the apparent spontaneous diffusion rate for an M13 particle in agarose (4.0E-13 m^2^/sec) is expected to be less than for AHL (7.1E-11 m^2^/sec), with a corresponding impact on expected average run lengths (<X > _M13_ = ~0.08 < X > _AHL_) (Additional file 2) [47,48]. We note that the AHL diffusion rate used here is ~4-fold greater than the widely-referenced value given in Basu et al. (1.7E-11 m^2^/sec, [8]) and that an increased rate seems better matched to reported results [49,50]. Meanwhile, most genetic messages of interest are likely to be encoded by DNA ranging from several hundred to many thousand base pairs. As a directly comparative example, LuxI, the enzyme that synthesizes 3OC_6_HSL (AHL) is encoded by a 579 bp open reading frame. If an expression cassette encoding for constitutive expression of LuxI were placed within a minimal messaging vector then a total of ~2,000 bp would be packaged, resulting in an M13 messaging particle with an expected diffusion rate of 1.0E-12 m^2^/sec, and thus an average daily run length ~8-fold less than AHL itself.

We therefore coupled M13-mediated messaging to bacterial chemotaxis in order to increase the length across which DNA messages can be transmitted. The native *E. coli* chemotaxis system can actively translocate cells ~10-30 microns per second across short distances, generating apparent random diffusion coefficients in liquid of 1-3E-10 m^2^/sec (<X > = 6–18 mm/day), and biased directional movement over several centimeters per day in gradients of chemo-attractants or -repellents [51]. The coupling of cell movement to cell-cell communication is widespread in natural systems that establish complex dynamic patterns, from fruiting body formation in myxobacteria [52] to angiogenesis in mammals [53]. We expect that engineered small molecule signaling systems can also be coupled to cell motility, along with DNA-based messaging, in order to provide engineers with a full suite of tools for programming dynamic patterns that span the microscopic to observable length scales (e.g., scaffold-free tissue engineering).

We also expect that the M13 system established here can be improved. First, conditional control of M13 gene product synthesis would allow for M13-mediated messaging to be regulated by external or intracellular signals. Second, additional messaging vectors spanning a range of DNA copy numbers would allow for control of load effects within transmitter cells, and the matching of message-encoded signal levels to genetic devices pre-existing within receiver cells. Third, a suite of variable strength M13 packaging sequences might enable control over the production of “broadcasting” M13 particles that contain the “helper phage” plasmid, encoding M13 gene products, enabling engineers to program the rate at which receiver cells convert to rebroadcasting cells capable of transmitting or amplifying messages. Fourth, past work to develop M13 particles capable of transducing mammalian cells could be adapted to enable autonomous trans-kingdom communication of DNA messages [54,55]. More broadly, the ongoing development and improvement of all types of engineered cell-cell communication platforms is needed to enable future bioengineers to create systems that match the richness of biological patterns and programs found in nature.

## Conclusions

We demonstrate the successful decoupling of a communication channel from message transmission within an engineered biological system via the autonomous targeted transduction of user-specified heterologous DNA messages. We also demonstrate that M13 particle production and transduction occurs among chemotactic bacteria. We use chemotaxis to increase the distance that DNA messages can travel, exceeding the transmission range and bit rate of existing small molecule-based communication systems. We postulate that integration of various engineered cell-cell communication platforms will allow for more complex spatial programming of dynamic cellular consortia.

## Materials and methods

### Strains and media

We conducted all co-culture experiments, except for lock-and-key messaging, in liquid culture using *E. coli* strain RP437 [42]. The lock-and-key co-culture experiment was conducted using *E. coli* strain XL1-Blue (Stratagene). We conducted experiments on agar plates using *E. coli* RP437, F^+^. RP437, F^+^ cells were created by mating RP437 with XL1-Blue via conjugation. We plated all transformations on LB agar supplemented with 10 μg/mL tetracycline, 30 μg/mL kanamycin, 50 μg/mL streptomycin, 25 μg/mL chloramphenicol, or 50 μg/mL carbenicillin, as necessary. We maintained clonal populations of transformants in glycerol stocks composed of 900 μL 60% sterile glycerol and 900 μL cell culture stored at -80C. We maintained liquid cultures of each cell type in 0.2 micron-filtered LB supplemented with appropriate antibiotics at 4C for no more than 5 days.

### Messaging phagemid and plasmid construction

We constructed and cloned two BioBrick-based messages (noted below) into a Litmus28i backbone using standard molecular biology techniques [56][,57]. We created additional constructs in a similar fashion to identify specific cell types within our system. We purchased all enzymes from New England Biolabs unless otherwise stated. Linearized Litmus28i was prepared by restriction digest using the enzymes EcoRI and PstI.

#### Litmus28i-I716104

We removed the BioBrick part I716104, which encodes the T7 RNA polymerase gene, from the BioBrick plasmid pSB1A2 by restriction digest using the enzymes EcoRI and PstI [57]. We then ligated the digested I716104 into linearized Litmus28i with T4 ligase and transformed the ligation into chemically-competent *E. coli* cells by heat shock.

#### Litmus28i-J23115-B0032-GFP

We added the composite part J23115-B0032 to GFP, BBa_E0040, in the BioBrick plasmid pSB1A2 with a single PCR using primers (1) and (2). The resulting PCR product was digested with restriction enzymes EcoRI and PstI. We then ligated the construct to linearized Litmus28i with T4 ligase and transformed the ligation into chemically-competent *E. coli* cells by heat shock.

#### pSB4C5-J64997-B0034-E0051

The original construction of *Gemini*, BBa_E0051, was described by Martin et al. [41]. We added a consensus T7 promoter (BioBrick part J64997) and ribosome binding site (BioBrick part B0034) in a single PCR reaction using primers (2) and (3). These primers also added the BioBrick prefix and suffix to the construct [57]. We digested the resulting construct with the restriction enzymes EcoRI and PstI. We ligated the digested construct into linearized BioBrick plasmid pSB4C5 (digested with EcoRI and PstI) with T4 ligase and transformed the ligation into chemically-competent *E. coli* cells by heat shock [57,58].

#### pSB4C5-J23119-RBS (C-dog)-mKate2

The mKate2 gene was synthesized by Blue Heron and cloned downstream of the constitutive BioBrick promoter J23119 and a strong RBS from the BioFAB C-dog pilot project onto a low copy BioBrick plasmid, pSB4C5 [58]. The resulting construct was transformed into chemically-competent *E. coli* cells by heat shock.

### Primers used

(1) 5’-TAC TAG GAA TTC GCG GCC GCT TCT AGA GTT TAT AGC TAG CTC AGC CCT TGG TAC AAT GCT AGC TAG TAG AG-3’

(2) 5’- CTG CAG CGG CCG CTA CTA GTA -3’

(3) 5’-GAA TTC GCG GCC GCT TCT AGA GTA ATA CGA CTC ACT ATA GGG TAC TAG ATG ACC ATG ATT ACG GAT TCA C-3’

### Preparation of sender and receiver cells

#### Sender cells

For all experiments conducted using *E. coli* strain RP437 or RP5838, we prepared infected sender cells by co-transforming chemically-competent cells with M13K07 phagemid in addition to the messaging phagemid, Litmus28i_J23115-B0034-GFP.

We prepared sender cells for the lock-and-key messaging experiment by diluting overnight cultures containing the T7 RNA polymerase messaging phagemid ten-fold into fresh LB media containing carbenicillin and tetracycline. We then added M13K07 phage (NEB) at a multiplicity of 5–10. We incubated the diluted cultures at 37C with shaking to return them to early log phase (OD_600_ ≈ 0.2). We then diluted the early log phase cultures ten-fold further into fresh LB media containing tetracycline, ampicillin, and kanamycin, and incubated at 37C with shaking overnight to near saturation.

#### Receiver cells

For the co-culture experiments, we transformed chemically-competent RP437 cells with the pSB4C5-J23119-RBS (C-dog)-mKate2 plasmid via heat shock. For the lock-and-key experiments, receiver cells were similarly transformed with the pSB4C5-J64997-B0034-E0051 construct. We cultured receiver cells in LB media containing tetracycline and chloramphenicol overnight with shaking at 37C. For use in the 2D message transmission experiments, we also transformed RP5838, F^+^ cells with the pSB4C5-J23119-RBS (C-dog)-mKate2 plasmid via heat shock.

### Liquid-based experiments

#### Dual-fluorescence experiments

Sender cells used in this portion of our study contained the Litmus28i-J23115-B0032-GFP message. Receiver cells contained the plasmid pSB4C5-J23119-RBS (C-dog)-mKate2. A positive control was constructed by co-transforming the pSB4C5-J23119-RBS (C-dog)-mKate2 and Litmus28i-J23115-B0032-GFP constructs into chemically-competent *E. coli* cells.

We diluted overnight cultures of sender, receiver, and positive control cells twenty-fold into fresh media with streptomycin. The diluted cultures were returned to log phase (OD_600_ ≈ 0.7) by incubation with shaking at 37C, after which we measured OD. From these log phase cultures we prepared four separate flasks of cells, each containing a total of 2×10^8^ cells in 20 mL fresh media with streptomycin. The first flask contained an equal proportion of sender and receiver cells; the second flask contained only sender cells; the third flask contained only receiver cells. Immediately following addition of the appropriate cells we gently swirled the flasks and removed a 500 μL aliquot of diluted culture. After removing an aliquot, we incubated all flasks at 37C with shaking for 5 hours and then removed another 500 μl aliquot. We immediately fixed each removed aliquot by addition of paraformaldehyde (PFA) (Electron Microscopy Sciences #15714-S) to a final concentration of 1% followed by storage at 4C. Fixed cells were analyzed using a 488 nm blue laser and 532 green laser on a BD LSR II FACS (Stanford FACS Core Facility); for each sample, we collected 30,000 events triggered on side scatter.

#### Lock-and-key experiments

Sender cells used in this portion of our study contained the Litmus28i-I716103 message. Receiver cells contained the BioBrick-based construct pSB4C5-J64997-B0034-E0051. A positive control was constructed by co-transforming the pSB4C5-J64997-B0034-E0051 and I716103-Litmus28i constructs into chemically-competent *E. coli* cells.

We diluted near-saturated cultures of both sender cells, receiver cells, and the positive control ten-fold into fresh media containing tetracycline. The diluted cultures were returned to early log phase (OD_600_ ≈ 0.2) by incubation with shaking at 37C. From these early log cultures we prepared six separate flasks of cells. The first three flasks were replicates and contained a twenty-fold dilution of each sender and receiver culture in fresh media containing tetracycline; the fourth flask contained a ten-fold dilution of sender cells in fresh media containing tetracycline; the fifth flask contained a ten-fold dilution of receiver cells in fresh media containing tetracycline; the sixth flask contained a ten-fold dilution of the positive control in fresh media containing tetracycline. Immediately following addition of the appropriate cells we gently swirled the flasks and removed a 500μL aliquot of diluted culture. We immediately fixed each removed aliquot by addition of paraformaldehyde (PFA) (Electron Microscopy Sciences #15714-S) to a final concentration of 1% followed by storage at 4C. We incubated the culture flasks at 37C with shaking for for 5 hours after which we added chloramphenicol and ampicillin to flasks containing co-cultures or the positive control, ampicillin to the flask containing sender cells, and chloramphenicol to the flask containing receiver cells. Following incubation at 37C with shaking to near saturation, we removed and fixed a second 500μL aliquot. Fixed cells were analyzed using a 488 nm blue laser on a BD LSR II FACS (Stanford FACS Core Facility); for each sample, we collected 30,000 events triggered on side scatter.

### 2D message transmission experiments

We prepared swim plates with 23 mL tryptone broth supplemented with 0.2% agar, 50 μg/ml streptomycin, and 10 μg/ml tetracycline. Sender cells—either motile RP437, F^+^ or non-motile RP5838, F^+^ cells—used in this experiment contained the J23115-B0032-GFP-Litmus28i message and M13K07 phagemid. Receiver cells—either motile RP437, F^+^ or non-motile RP5838, F^+^ cells—contained the J23119-RBS (C-dog)-mKate2-pSB4C5 plasmid.

We inoculated swim plates with near-saturated cultures of the sender and receiver cells by spotting 1 μl of sender cell culture 1 cm from the left edge of the plate and 1 μl of receiver cell culture 1 cm from the right edge of the plate. We incubated all plates face up at 37C with humidity (provided by sponges soaking in DI water) for 48 hours. Plates were imaged within a black box for GFP fluorescence (excitation 490–510 nm; emission 520–540 nm) and RFP fluorescence (excitation 600–620 nm; emission 630–660 nm). Bright field images were also collected for each plate, and the fluorescence and bright field images were merged.

For the swim plate with both motile sender and receiver cells, we scraped the surface of the collision region and directly imaged these cells on an agarose pad via light and fluorescence microscopy. We also removed 9 equally spaced agar plugs from each plate along a horizontal line connecting the inoculation sites of the sender cells and receiver cells using 1.1 mm × 6.5 mm gel extraction tips (Phenix). We briefly resuspended each agar plug in 200 μl LB liquid media and then pipetted 10 μl of each resuspension into 1 mL LB liquid media containing ampicillin, chloramphenicol, or both ampicillin and chloramphenicol. These cultures were incubated with shaking at 30C for 24 hours.

After incubation, we transferred a 200 μl aliquot of each culture into a flat-bottomed 96-well plate (Nunc) and measured the OD_600_ of each culture on a Wallac Victor3 multi-well fluorimeter (Perkin Elmer). In addition, we imaged cells from these resulting cultures for GFP and RFP fluorescence (using 50 ms and 200 ms exposure times, respectively) and determined background fluorescence from monocultures of sender cells or receiver cells.

## Competing interests

The authors declare that they have no competing interests.

## Authors' contributions

MEO and DE designed the experiments. MEO carried out the experiments. MEO and DE interpreted the data, and wrote and approved the manuscript. Both authors read and approved the final manuscript.

## Supplementary Material

Additional file 1 Supplemental Calculations. Click here for file

Additional file 2 Construct Sequences. Click here for file
