# Supplementary material for: Engineered cell-cell communication via DNA messaging
Source: J Biol Eng. 2012 Sep 7;6:16. doi: 10.1186/1754-1611-6-16 (PMC3509006; doi:10.1186/1754-1611-6-16)

## I. Supplemental Calculations

***Diffusion analysis for AHL- and bacteriophage M13-based cell-cell communication systems:***

Hu et al. calculated the diffusion coefficient of fNEL, a filamentous bacteriophage with dimensions 1300 nm x 15 nm, to be 2.6 x $10^{-13}$ $m^2/s$ [Ref. 58]. Modeling the bacteriophage as a rod, we calculated the diffusion coefficient for a bacteriophage M13 particle traveling through agarose along its major axis by finding the viscosity coefficient of agarose, then calculating the diffusion coefficient using the dimensions of a bacteriophage M13 particle, 930 nm x 6 nm (Eqs. 1-4) [Ref. 59]. We calculated the diffusion coefficient for a bacteriophage M13 particle to be 4.04 x $10^{-13}$ $m^2/s$.

$$D_{app,FNEL} = \frac{kT * ln(\frac{2a}{b})}{6\pi\eta a} = 2.6 * 10^{-13} m^2/s \quad (1)$$

$$D_{app,FNEL} = \frac{(1.38 * 10^{-23} J * K^{-1})(310.15K) * ln(\frac{2(650nm)}{7.5nm})}{6\pi\eta(650nm)} = 2.6 * 10^{-13} m^2/s \quad (2)$$

$$\eta = 6.93 * 10^{-3} Pa * s \quad (3)$$

$$D_{M13} = \frac{(1.38 * 10^{-23} J * K^{-1})(310.15K) * ln(\frac{2(465nm)}{3nm})}{6\pi\eta(465nm)} = 4.04 * 10^{-13} m^2/s \quad (4)$$

Next, we back-calculated the effective radius for N-(3-Oxododecanoyl)-L-homoserine lactone from its diffusion constant calculated in water at 22C. We then calculated the diffusion constant for the same through agar at 37C [47,48].

$$D_{AHL,water,22C} = 4.9 * 10^{-10} m^2/s = \frac{k(295.15K)}{6\pi * 0.95 mPa \cdot s * r_{effective}} \quad (5)$$

$$r_{effective} \approx 0.46nm \quad (6)$$

We used the viscosity for agarose used for the M13 diffusion coefficient calculation (Eq. 3-4) and the effective radius found above (Eq. 6) to find the diffusion coefficient at 37C through agarose (Eq. 7).

$$D_{AHL,agar,37C} \approx \frac{k * 310.15K}{6\pi(6.93 * 10^{-3}Pa \cdot s)(0.46 * 10^{-9}m)} = 7.1 * 10^{-11}m^2/s \tag{7}$$

We used the diffusion coefficients to calculate the root mean squared (RMS) distances in two dimensions for both AHL and bacteriophage M13 over some time, Δt. We also calculated the ratio between the root mean squared distances of AHL and bacteriophage M13 (Eqs. 8-11).

$$RMS = \sqrt{< x^2 >} = \sqrt{4D\Delta t} \tag{8}$$

$$RMS_{M13} = \sqrt{4D_{M13}\Delta t} \approx \sqrt{4(4.04 * 10^{-13}m^2/s)\Delta t} = (1.27 * 10^{-6} * \sqrt{\Delta t})m \tag{9}$$

$$RMS_{AHL} = \sqrt{4D_{AHL}\Delta t} \approx \sqrt{4(7.1 * 10^{-11}m^2/s)\Delta t} = (1.7 * 10^{-5} * \sqrt{\Delta t})m \tag{10}$$

$$\frac{RMS_{AHL}}{RMS_{M13}} = \frac{1.7 * 10^{-5}}{1.3 * 10^{-6}} \approx 13 \tag{11}$$

***Effective range of a DNA message transmission coupled to bacterial chemotaxis:***

The speed of a DNA message within our bacteriophage M13-based cell-cell communication platform increases when we couple message transmission to bacterial chemotaxis. Referring to Figure 4 in the main text, we demonstrated message transmission at 3.5 cm from an seed spot of sender cells within 48 hours.

Estimating the velocity of cells at the collision region in the center of the plate, we have:

$$\overline{V_{motility}} = \frac{3.5cm}{48hr \cdot \frac{1day}{24hr}} \approx 1.8\frac{cm}{day} \tag{12}$$

Calculating the distance a DNA message travels after 1 day without the aid of cell motility, we have:

$$RMS_{M13\ diffusion} = 1.27 * 10^{-6}\frac{m}{\sqrt{s}} * \sqrt{86400s} \approx 0.37mm \tag{13}$$

Thus, we estimate that the increase in effective speed of a DNA message within our M13-based system while coupled to bacterial chemotaxis is:

$$\frac{Distance_{Motility}}{Distance_{Diffusion}} \approx \frac{1.8cm/day}{0.37mm/day} = 49\ fold\ increase \tag{14}$$

## II. Supplemental Figures

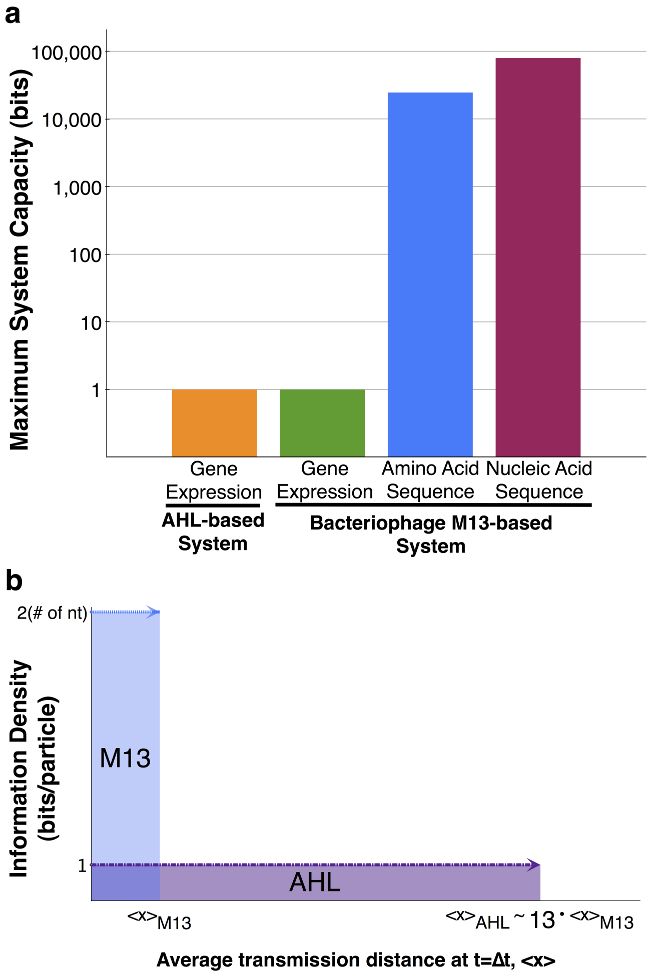


**Supp. Fig. 1: Channel capacity and transmission distances differ between AHL- and M13-based cell-cell communication systems.** (a) AHL-based systems are used to control activation of transcription and can be turned ON or OFF (1 bit). M13-based systems can be used to transmit any genetically encoded message below the maximum phage particle length; as such, M13-based systems can encode messages equivalent to AHL (1 bit) and also higher-information content messages such as peptide and nucleic acid sequences (values are calculated for a 6400 nucleotide message). (b) M13-based systems transmit more information but over a shorter range than AHL-based systems with diffusion alone. Theoretical projections of M13 and AHL information transmission density maps. Distance traveled is shown over an arbitrary time period, $\Delta t$, during which a M13 particle travels $\langle x\rangle_{M13}$ while an AHL signal molecule travels ~13-fold further, $\langle x\rangle_{AHL}$. We note that methods to increase the range of a DNA message within the M13-based system, e.g., coupling to chemotaxis, increase the effective transmission distance.

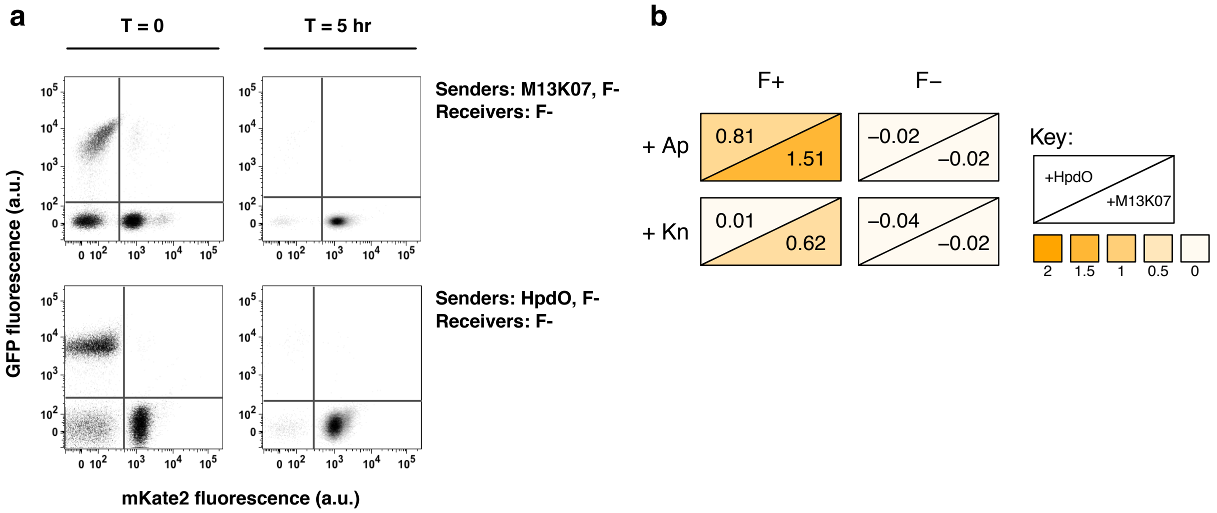


**Supp. Fig. 2: M13-based cell-cell communication does not occur in absence of the F-plasmid.** (a) *Co-cultures of $F^-$ sender and receiver cells cannot transmit a DNA message.* We prepared $F^-$ sender cells transduced with M13K07 or HpdO, M13 helper phages with or without the M13 packaging sequence, respectively, by co-transforming each helper phage and message phagemid, Litmus28i_J23115-B0034-GFP, into chemically competent RP437, $F^-$ cells via heat shock. We prepared $F^-$ receiver cells by transforming chemically-competent RP437, $F^-$ cells with the pSB4C5-J23119-RBS (C-dog)-mKate2 plasmid via heat shock. Co-cultures were prepared and analyzed as presented in the main text. Flow cytometry plots show mKate2 fluorescence (x-axis) versus GFP fluorescence (y-axis). Fluorescence data of GFP and mKate2 are shown for co-cultures at the start of an experiment (T = 0) and after 5 hours of co-culture without antibiotic selection (T = 5 hr). (b) *Co-cultures of $F^-$ sender and receiver cells contain message particles, which can transmit message to $F^+$ receiver cells.* For each co-culture described above, we passed supernatant from each through a 0.2 micron filter (Pall). To mid-log phase $F^-$ and $F^+$ receiver cells, we added 10μl of the filtered supernatant and incubated each culture at 37C with shaking for 1 hour. We subsequently diluted each culture into fresh media containing either ampicillin or kanamycin and grew these cultures for 24 hours at 30C with shaking. We then measured OD of each culture as described in the main text. Color bar at right indicates color saturation for specified OD values, also indicated numerically within each triangle. Data points within upper triangles were collected from cultures to which we added HpdO-sender cell co-culture filtrate. Data points from lower triangles were collected from cultures to which we added M13K07-sender cell co-culture filtrate.

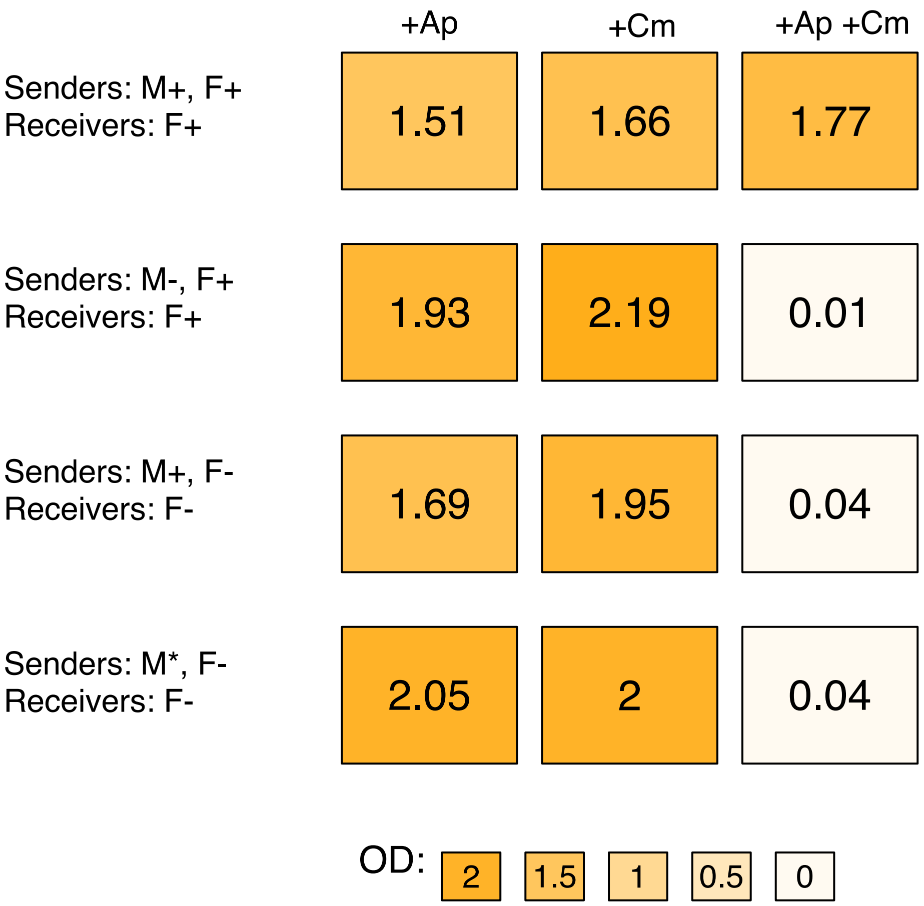


**Supp. Fig. 3: Absorbance of co-cultures under antibiotic selection.** OD measurements of co-cultures diluted into fresh media containing various antibiotics. Color bar at bottom indicates color saturation for specified OD values, also labeled numerically within each box.

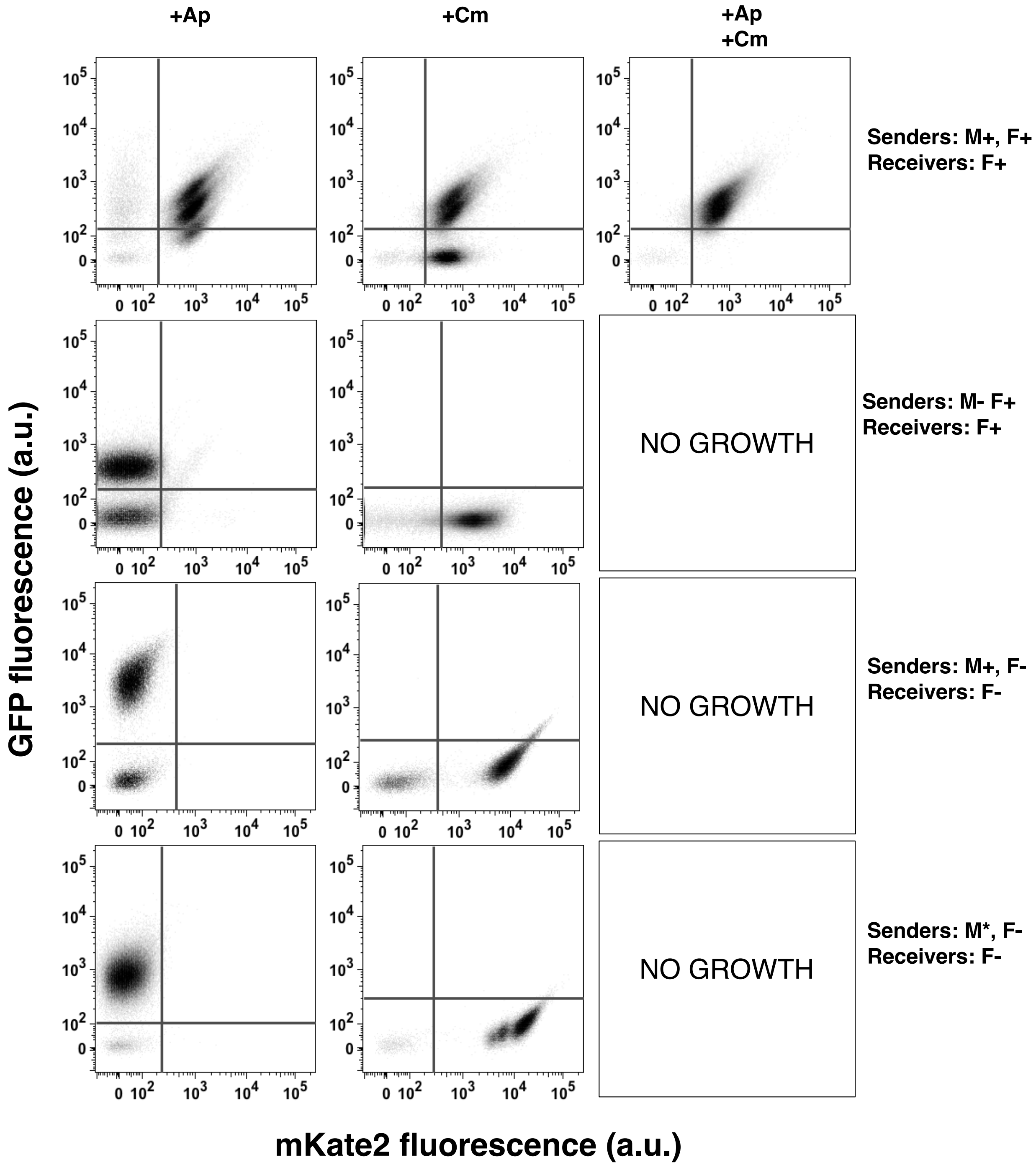


**Supp. Fig. 4: Flow cytometry of co-cultures under antibiotic selection.** Flow cytometry analysis of antibiotic-containing subcultures of co-cultures. Flow cytometry plots show mKate2 fluorescence (x-axis) versus GFP fluorescence (y-axis). Antibiotics present in media are documented at the top of the plot and genotypes of sender and receiver cells are found at the right. "M" denotes presence or absence of M13K07 helper phage, and "M*" denotes presence of HpdO helper phage. "F" denotes presence or absence of the F-plasmid.

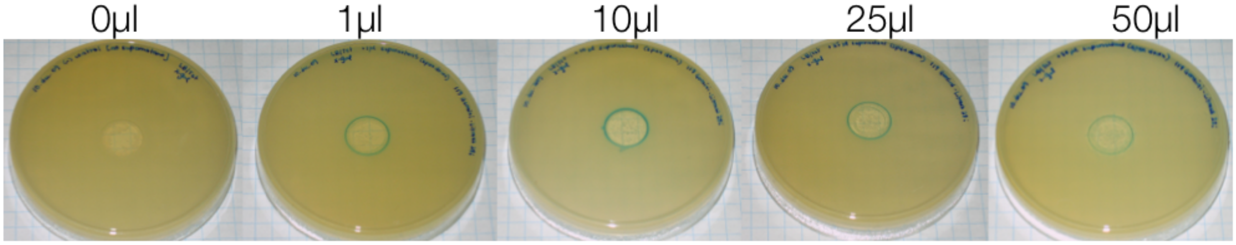


**Supp. Fig. 5: Activation of beta-galactosidase activity in receiver cells via message particle diffusion alone.** Mid-log growth phase XL1-Blue E. *coli* (Stratagene) were prepared by diluting an overnight culture into fresh media containing 10 µg/ml tetracycline and incubating at 37C with shaking until OD600 ~ 0.6. To LB with 0.6% agar held at 48C, we added 200µl of mid-log XL1-Blue cells, as well as 0.1M IPTG (Sigma-Aldrich) and 25 mg/ml X-gal (Sigma-Aldrich). The contents were vortexed, then poured onto a LB agar plate around a sterile test tube cap at the center of the plate, thus forming a small well. The top agar was allowed to solidify at room temperature for 20 minutes, after which the test tube cap was removed. From an overnight culture of XL1-Blue cells transformed with M13K07 and Litmus_J23119-B0032-E0051, message particles present in the supernatant were isolated via filtration as described above. To the wells of plates with XL1-Blue-containing top agar, we added {0, 1, 10, 25, 50}µl to sterile water to a total of 50 µl. Plates were incubated for 18 hours at 37C, after which they were imaged with a Panasonic Lumix DMC-TZ3 digital camera (shown here). Values above each photograph indicate the volume of supernatant added to each plate. Construction of Litmus28i_J23119-B0032-E0051 is described as follows. We digested the composite BioBrick part J23119-B0032-E0051, originally described in Martin et al. (Ref. 41), with the restriction enzymes EcoRI and PstI. This reaction excised the J23119 promoter, B0032 ribosome binding site, E0051 gene from the pSB1A3 plasmid. We then ligated the construct to linearized Litmus28i with T4 ligase and transformed the ligation into chemically-competent *E. coli* cells by heat shock. Specific sequences are freely available via http://partsregistry.org/.

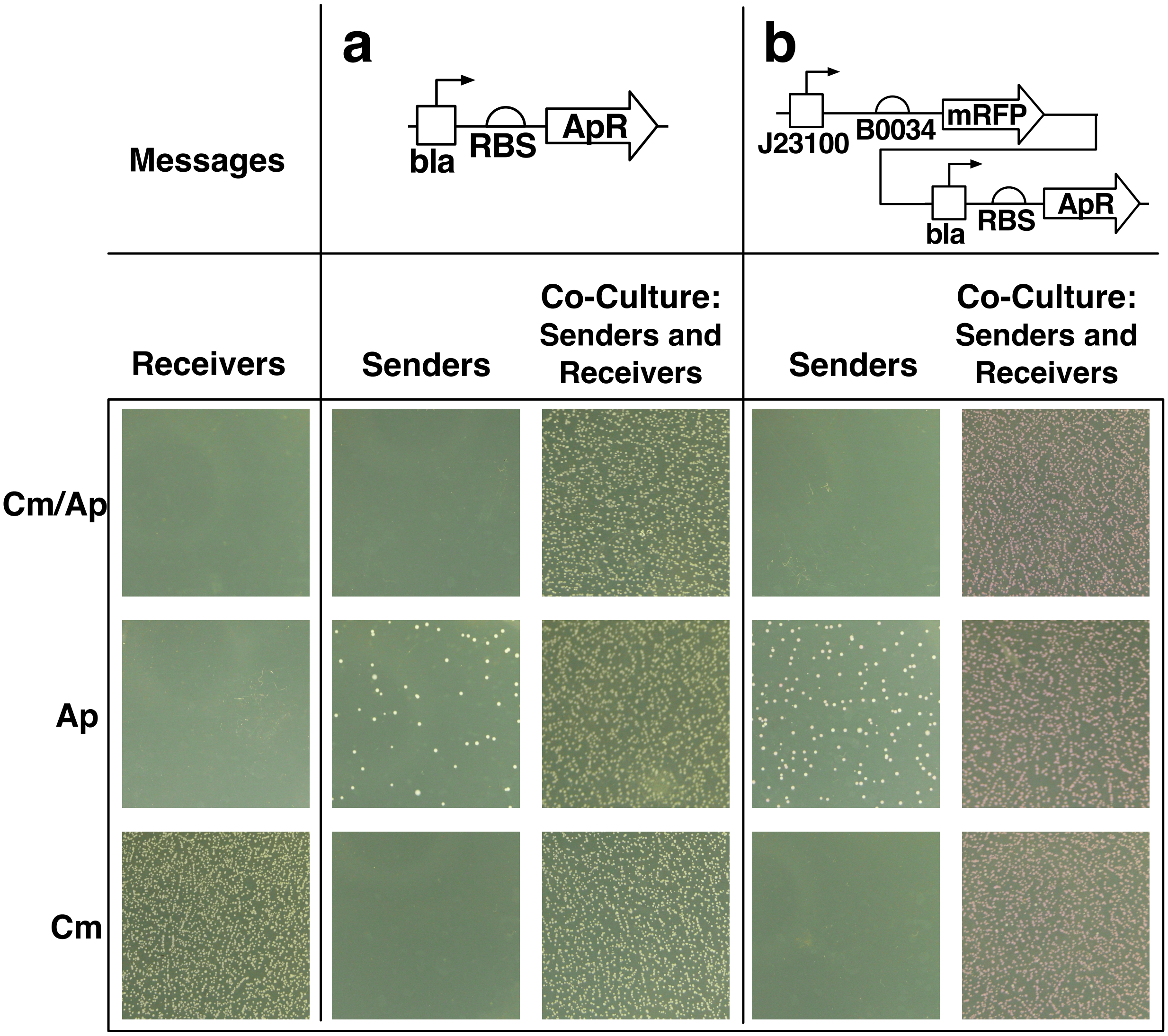


**Supp. Fig. 6: Additional DNA messages sent via M13-based cell-cell communication.** Photographs show representative plates of sender or receiver monocultures, or co-cultures (methods as described in the main text) plated onto semi-solid media containing antibiotics as indicated at left. (a) Sender cells contain and transmit "ampicillin resistance" via transmission of the Litmus28i phagemid without modification. Sender cells were co-transformed with Litmus28i phagemid and M13K07 helper phage (New England Biolabs). Co-cultures were performed as described in the main text. (b) As in (a), but sender cells encode and transmit "ampicillin resistance AND red fluorescent protein" via transmission of Litmus28i containing a constitutively-expressed RFP construct. We digested the composite BioBrick part J23100-J61002 with the restriction enzymes EcoRI and PstI. This reaction excised the J23100 promoter, B0034 ribosome binding site, mRFP1 gene, and B0015 terminator from the J61002 plasmid. We then ligated the construct to linearized Litmus28i with T4 ligase and transformed the ligation into chemically-competent *E. coli* cells by heat shock. The specific sequence for J23110-mRFP1 is freely available via http://partsregistry.org/. Receiver cells are chloramphenicol resistant.

# III. Annotated construct maps

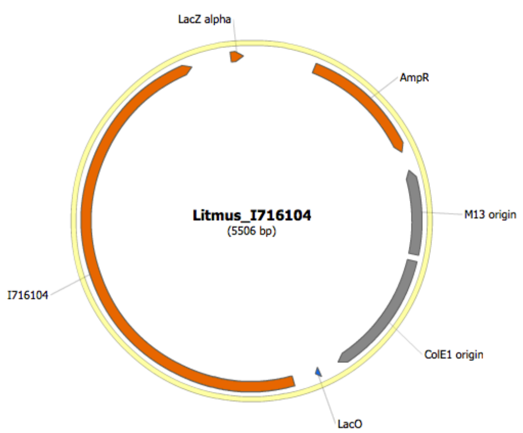


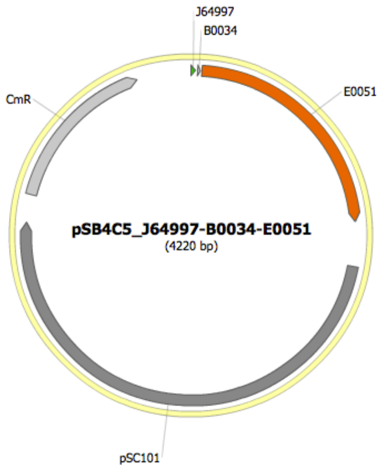


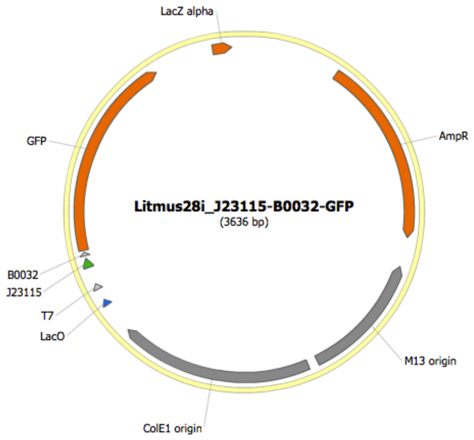


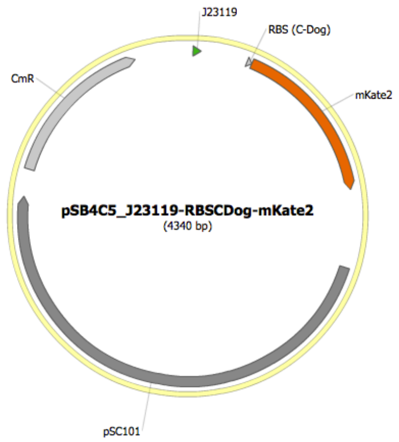


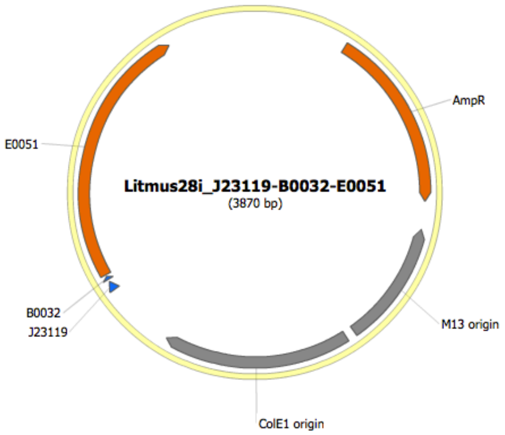

Supplement: Additional file 1 — Supplemental Calculations. [file 1754-1611-6-16-S1.pdf]
