# Supplementary material for: Engineered cell-cell communication via DNA messaging
Source: J Biol Eng. 2012 Sep 7;6:16. doi: 10.1186/1754-1611-6-16 (PMC3509006; doi:10.1186/1754-1611-6-16)
Supplement: Additional file 2 — Construct Sequences. [file 1754-1611-6-16-S2.pdf]

IV. Construct Sequences

```
Thu Jan 19, 2012 11:39 PST
I716104-Litmus28i.ape
Text Map
             10        20        30        40        50        60        70        80        90       100
         *         *         *         *         *         *         *         *         *         *
   1 gttaactacgtcaggtggcacttttcggggaaatgtgcgcggaacccctatttgtttatttttctaaatacattcaaatatgtatccgctcatgagacaa 100

            110       120       130       140       150       160       170       180       190       200
         *         *         *         *         *         *         *         *         *         *
 101 taaccctgataaatgcttcaataatattgaaaaaggaagagtatgagtattcaacatttccgtgtcgcccttattcccttttttgcggcattttgccttc 200

            210       220       230       240       250       260       270       280       290       300
         *         *         *         *         *         *         *         *         *         *
 201 ctgtttttgctcacccagaaacgctggtgaaagtaaaagatgctgaagatcagttgggtgcacgagtgggttacatcgaactggatctcaacagcggtaa 300

            310       320       330       340       350       360       370       380       390       400
         *         *         *         *         *         *         *         *         *         *
 301 gatccttgagagttttcgccccgaagaacgttctccaatgatgagcacttttaaagttctgctatgtggcgcggtattatcccgtgttgacgccgggcaa 400
                                             M  S  T  F  K  V  L  L  C  G  A  V  L  S  R  V  D  A  G  Q
                                             >>>>>>>>>>>>>>>>>>>>>>>>>>>>>>>>>>>>>>>>>>>>>>>>>>>>>>>>>>>>
                                             AmpR

            410       420       430       440       450       460       470       480       490       500
         *         *         *         *         *         *         *         *         *         *
 401 gagcaactcggtcgccgcatacactattctcagaatgacttggttgagtactcaccagtcacagaaaagcatcttacggatggcatgacagtaagagaat 500
     E  Q  L  G  R  R  I  H  Y  S  Q  N  D  L  V  E  Y  S  P  V  T  E  K  H  L  T  D  G  M  T  V  R  E  L
     >>>>>>>>>>>>>>>>>>>>>>>>>>>>>>>>>>>>>>>>>>>>>>>>>>>>>>>>>>>>>>>>>>>>>>>>>>>>>>>>>>>>>>>>>>>>>>>>>>>>
     AmpR

            510       520       530       540       550       560       570       580       590       600
         *         *         *         *         *         *         *         *         *         *
 501 tatgcagtgctgccataaccatgagtgataacactgcggccaacttacttctgacaacgatcggaggaccgaaggagctaaccgcttttttgcacaacat 600
       C  S  A  A  I  T  M  S  D  N  T  A  A  N  L  L  L  T  T  I  G  G  P  K  E  L  T  A  F  L  H  N  M
     >>>>>>>>>>>>>>>>>>>>>>>>>>>>>>>>>>>>>>>>>>>>>>>>>>>>>>>>>>>>>>>>>>>>>>>>>>>>>>>>>>>>>>>>>>>>>>>>>>>>
     AmpR

            610       620       630       640       650       660       670       680       690       700
         *         *         *         *         *         *         *         *         *         *
 601 ggggatcatgtaactcgccttgatcgttgggaaccggagctgaatgaagccataccaaacgacgagcgtgacaccacgatgcctgtagcaatggcaaca 700
      G  D  H  V  T  R  L  D  R  W  E  P  E  L  N  E  A  I  P  N  D  E  R  D  T  T  M  P  V  A  M  A  T
     >>>>>>>>>>>>>>>>>>>>>>>>>>>>>>>>>>>>>>>>>>>>>>>>>>>>>>>>>>>>>>>>>>>>>>>>>>>>>>>>>>>>>>>>>>>>>>>>>>>>
     AmpR

            710       720       730       740       750       760       770       780       790       800
         *         *         *         *         *         *         *         *         *         *
 701 acgttgcgcaaactattaactggcgaactacttactctagcttcccggcaacaattaatagactggatggaggcggataaagttgcaggaccacttctgc 800
     T  L  R  K  L  L  T  G  E  L  L  T  L  A  S  R  Q  Q  L  I  D  W  M  E  A  D  K  V  A  G  P  L  L  R
     >>>>>>>>>>>>>>>>>>>>>>>>>>>>>>>>>>>>>>>>>>>>>>>>>>>>>>>>>>>>>>>>>>>>>>>>>>>>>>>>>>>>>>>>>>>>>>>>>>>>
     AmpR

            810       820       830       840       850       860       870       880       890       900
         *         *         *         *         *         *         *         *         *         *
 801 gctcggcccttccggctggctggtttattgctgataaatctggagccggtgagcgtgggtctcgcggtatcattgcagcactggggccagatggtaagcc 900
       S  A  L  P  A  G  W  F  I  A  D  K  S  G  A  G  E  R  G  S  R  G  I  I  A  A  L  G  P  D  G  K  P
     >>>>>>>>>>>>>>>>>>>>>>>>>>>>>>>>>>>>>>>>>>>>>>>>>>>>>>>>>>>>>>>>>>>>>>>>>>>>>>>>>>>>>>>>>>>>>>>>>>>>
     AmpR

            910       920       930       940       950       960       970       980       990      1000
         *         *         *         *         *         *         *         *         *         *
 901 ctcccgtatcgtagttatctacacgacggggagtcaggcaactatggatgaacgaaatagacagatcgctgagataggtgcctcactgattaagcattgg 1000
      S  R  I  V  V  I  Y  T  T  G  S  Q  A  T  M  D  E  R  N  R  Q  I  A  E  I  G  A  S  L  I  K  H  W
     >>>>>>>>>>>>>>>>>>>>>>>>>>>>>>>>>>>>>>>>>>>>>>>>>>>>>>>>>>>>>>>>>>>>>>>>>>>>>>>>>>>>>>>>>>>>>>>>>>>>
     AmpR

           1010      1020      1030      1040      1050      1060      1070      1080      1090      1100
         *         *         *         *         *         *         *         *         *         *
```

```
1001 taactgtcagaccaagtttactcatatatactttagattgatttaccccggttgataatcagaaaagccccaaaaacaggaagattgtataagcaaatat 1100

         1110      1120      1130      1140      1150      1160      1170      1180      1190      1200
        *         *         *         *         *         *         *         *         *         *
1101 ttaaattgtaaacgttaatattttgttaaaattcgcgttaaatttttgttaaatcagctcattttttaaccaataggccgaaatcggcaaaatcccttat 1200
       <<<<<<<<<<<<<<<<<<<<<<<<<<<<<<<<<<<<<<<<<<<<<<<<<<<<<<<<<<<<<<<<<<<<<<<<<<<<<<<<<<<<<<<<<<<<<<<<<<
       M13 origin

         1210      1220      1230      1240      1250      1260      1270      1280      1290      1300
        *         *         *         *         *         *         *         *         *         *
1201 aaatcaaaagaatagcccgagatagggttgagtgttgttccagtttggaacaagagtccactattaaagaacgtggactccaacgtcaaagggcgaaaaa 1300
     <<<<<<<<<<<<<<<<<<<<<<<<<<<<<<<<<<<<<<<<<<<<<<<<<<<<<<<<<<<<<<<<<<<<<<<<<<<<<<<<<<<<<<<<<<<<<<<<<<<<
     M13 origin

         1310      1320      1330      1340      1350      1360      1370      1380      1390      1400
        *         *         *         *         *         *         *         *         *         *
1301 ccgtctatcagggcgatggcccactacgtgaaccatcacccaaatcaagttttttggggtcgaggtgccgtaaagcactaaatcggaaccctaaagggag 1400
     <<<<<<<<<<<<<<<<<<<<<<<<<<<<<<<<<<<<<<<<<<<<<<<<<<<<<<<<<<<<<<<<<<<<<<<<<<<<<<<<<<<<<<<<<<<<<<<<<<<<
     M13 origin

         1410      1420      1430      1440      1450      1460      1470      1480      1490      1500
        *         *         *         *         *         *         *         *         *         *
1401 cccccgatttagagcttgacggggaaagcgaacgtggcgagaaaggaagggaagaaagcgaaaggagcgggcgctagggcgctggcaagtgtagcggtca 1500
     <<<<<<<<<<<<<<<<<<<<<<<<<<<<<<<<<<<<<<<<<<<<<<<<<<<<<<<<<<<<<<<<<<<<<<<<<<<<<<<<<<<<<<<<<<<<<<<<<<<<
     M13 origin

         1510      1520      1530      1540      1550      1560      1570      1580      1590      1600
        *         *         *         *         *         *         *         *         *         *
1501 cgctgcgcgtaaccaccacacccgccgcgcttaatgcgccgctacagggcgcgtaaaaggatctaggtgaagatcctttttgataatctcatgaccaaaa 1600
                                                                                          >>>>>>>>>>>
                                                                                          ColE1 origin

     <<<<<<<<<<<<<<<<<<<<<<<<<<<<<<<<<<<<<<<<<<<<<<<<<<<<<<<
     M13 origin

         1610      1620      1630      1640      1650      1660      1670      1680      1690      1700
        *         *         *         *         *         *         *         *         *         *
1601 tcccttaacgtgagttttcgttccactgagcgtcagaccccgtagaaaagatcaaaggatcttcttgagatcctttttttctgcgcgtaatctgctgctt 1700
     >>>>>>>>>>>>>>>>>>>>>>>>>>>>>>>>>>>>>>>>>>>>>>>>>>>>>>>>>>>>>>>>>>>>>>>>>>>>>>>>>>>>>>>>>>>>>>>>>>>>
     ColE1 origin

         1710      1720      1730      1740      1750      1760      1770      1780      1790      1800
        *         *         *         *         *         *         *         *         *         *
1701 gcaaacaaaaaaaccaccgctaccagcggtggtttgtttgccggatcaagagctaccaactctttttccgaaggtaactggcttcagcagagcgcagata 1800
     >>>>>>>>>>>>>>>>>>>>>>>>>>>>>>>>>>>>>>>>>>>>>>>>>>>>>>>>>>>>>>>>>>>>>>>>>>>>>>>>>>>>>>>>>>>>>>>>>>>>
     ColE1 origin

         1810      1820      1830      1840      1850      1860      1870      1880      1890      1900
        *         *         *         *         *         *         *         *         *         *
1801 ccaaatactgttcttctagtgtagccgtagttaggccaccacttcaagaactctgtagcaccgcctacatacctcgctctgctaatcctgttaccagtgg 1900
     >>>>>>>>>>>>>>>>>>>>>>>>>>>>>>>>>>>>>>>>>>>>>>>>>>>>>>>>>>>>>>>>>>>>>>>>>>>>>>>>>>>>>>>>>>>>>>>>>>>>
     ColE1 origin

         1910      1920      1930      1940      1950      1960      1970      1980      1990      2000
        *         *         *         *         *         *         *         *         *         *
1901 ctgctgccagtggcgataagtcgtgtcttaccgggttggactcaagacgatagttaccggataaggcgcagcggtcgggctgaacggggggttcgtgcac 2000
     >>>>>>>>>>>>>>>>>>>>>>>>>>>>>>>>>>>>>>>>>>>>>>>>>>>>>>>>>>>>>>>>>>>>>>>>>>>>>>>>>>>>>>>>>>>>>>>>>>>>
     ColE1 origin

         2010      2020      2030      2040      2050      2060      2070      2080      2090      2100
        *         *         *         *         *         *         *         *         *         *
2001 acagcccagcttggagcgaacgacctacaccgaactgagatacctacagcgtgagctatgagaaagcgccacgcttcccgaagggagaaaggcggacagg 2100
     >>>>>>>>>>>>>>>>>>>>>>>>>>>>>>>>>>>>>>>>>>>>>>>>>>>>>>>>>>>>>>>>>>>>>>>>>>>>>>>>>>>>>>>>>>>>>>>>>>>>
     ColE1 origin

         2110      2120      2130      2140      2150      2160      2170      2180      2190      2200
        *         *         *         *         *         *         *         *         *         *
```

```
2101 tatccggtaagcggcagggtcggaacaggagagcgcacgagggagcttccagggggaaacgcctggtatctttatagtcctgtcgggtttcgccacctct 2200
     >>>>>>>>>>>>>>>>>>>>>>>>>>>>>>>>>>>>>>>>>>>>>>>>>>>>>>>>>>>>>>>>>>>>>>>>>>>>>>>>>>>>>>>>>>>>>>>>>>>>
     ColE1 origin

            2210      2220      2230      2240      2250      2260      2270      2280      2290      2300
        *         *         *         *         *         *         *         *         *         *
2201 gacttgagcgtcgatttttgtgatgctcgtcaggggggcggagcctatggaaaaacgccagcaacgcggcctttttacggttcctggccttttgctggcc 2300
     >>>>>>>>>>>>>>>>>>>>>>>>>>>>>>>>>>>>>>>>>>>>>>>>>>>>>>>>>>>>>>>>>>>>>>>
     ColE1 origin

            2310      2320      2330      2340      2350      2360      2370      2380      2390      2400
        *         *         *         *         *         *         *         *         *         *
2301 ttttgctcacatgtaatgtgagttagctcactcattaggcaccccaggctttacactttatgcttccggctcgtatgttgtgtggaattgtgagcggata 2400
                                                                                     >>>>>>>>>>>>>>>>>
                                                                                     LacO

            2410      2420      2430      2440      2450      2460      2470      2480      2490      2500
        *         *         *         *         *         *         *         *         *         *
2401 acaatttcacacaggaaacagctatgaccatgattacgccaagctacgtaatacgactcactatagggcagatcttcgaatgcatcgcgcgcaccgtacg 2500
     >>>>>>
     LacO

            2510      2520      2530      2540      2550      2560      2570      2580      2590      2600
        *         *         *         *         *         *         *         *         *         *
2501 tctcgaggaattcgcggccgcttctagaggtgaacacgattaacatcgctaagaacgacttctctgacatcgaactggctgctatcccgttcaacactct 2600
                                 V  N  T  I  N  I  A  K  N  D  F  S  D  I  E  L  A  A  I  P  F  N  T  L
                                 >>>>>>>>>>>>>>>>>>>>>>>>>>>>>>>>>>>>>>>>>>>>>>>>>>>>>>>>>>>>>>>>>>>>>
                                 I716104

            2610      2620      2630      2640      2650      2660      2670      2680      2690      2700
        *         *         *         *         *         *         *         *         *         *
2601 ggctgaccattacggtgagcgtttagctcgcgaacagttggcccttgagcatgagtcttacgagatgggtgaagcacgcttccgcaagatgtttgagcgt 2700
      A  D  H  Y  G  E  R  L  A  R  E  Q  L  A  L  E  H  E  S  Y  E  M  G  E  A  R  F  R  K  M  F  E  R
     >>>>>>>>>>>>>>>>>>>>>>>>>>>>>>>>>>>>>>>>>>>>>>>>>>>>>>>>>>>>>>>>>>>>>>>>>>>>>>>>>>>>>>>>>>>>>>>>>>>>
     I716104

            2710      2720      2730      2740      2750      2760      2770      2780      2790      2800
        *         *         *         *         *         *         *         *         *         *
2701 caacttaaagctggtgaggttgcggataacgctgccgccaagcctctcatcactaccctactccctaagatgattgcacgcatcaacgactggtttgagg 2800
     Q  L  K  A  G  E  V  A  D  N  A  A  A  K  P  L  I  T  T  L  L  P  K  M  I  A  R  I  N  D  W  F  E  E
     >>>>>>>>>>>>>>>>>>>>>>>>>>>>>>>>>>>>>>>>>>>>>>>>>>>>>>>>>>>>>>>>>>>>>>>>>>>>>>>>>>>>>>>>>>>>>>>>>>>>
     I716104

            2810      2820      2830      2840      2850      2860      2870      2880      2890      2900
        *         *         *         *         *         *         *         *         *         *
2801 aagtgaaagctaagcgcggcaagcgcccgacagccttccagttcctgcaagaaatcaagccggaagccgtagcgtacatcaccattaagaccactctggc 2900
       V  K  A  K  R  G  K  R  P  T  A  F  Q  F  L  Q  E  I  K  P  E  A  V  A  Y  I  T  I  K  T  T  L  A
     >>>>>>>>>>>>>>>>>>>>>>>>>>>>>>>>>>>>>>>>>>>>>>>>>>>>>>>>>>>>>>>>>>>>>>>>>>>>>>>>>>>>>>>>>>>>>>>>>>>>
     I716104

            2910      2920      2930      2940      2950      2960      2970      2980      2990      3000
        *         *         *         *         *         *         *         *         *         *
2901 ttgcctaaccagtgctgacaatacaaccgttcaggctgtagcaagcgcaatcggtcggccattgaggacgaggctcgcttcggtcgtatccgtgacctt 3000
      C  L  T  S  A  D  N  T  T  V  Q  A  V  A  S  A  I  G  R  A  I  E  D  E  A  R  F  G  R  I  R  D  L
     >>>>>>>>>>>>>>>>>>>>>>>>>>>>>>>>>>>>>>>>>>>>>>>>>>>>>>>>>>>>>>>>>>>>>>>>>>>>>>>>>>>>>>>>>>>>>>>>>>>>
     I716104

            3010      3020      3030      3040      3050      3060      3070      3080      3090      3100
        *         *         *         *         *         *         *         *         *         *
3001 gaagctaagcacttcaagaaaaacgttgaggaacaactcaacaagcgcgtagggcacgtctacaagaaagcatttatgcaagttgtcgaggctgacatgc 3100
     E  A  K  H  F  K  K  N  V  E  E  Q  L  N  K  R  V  G  H  V  Y  K  K  A  F  M  Q  V  V  E  A  D  M  L
     >>>>>>>>>>>>>>>>>>>>>>>>>>>>>>>>>>>>>>>>>>>>>>>>>>>>>>>>>>>>>>>>>>>>>>>>>>>>>>>>>>>>>>>>>>>>>>>>>>>>
     I716104

            3110      3120      3130      3140      3150      3160      3170      3180      3190      3200
        *         *         *         *         *         *         *         *         *         *
```

```
3101 tctctaagggtctactcggtggcgaggcgtggtcttcgtggcataaggaagactctattcatgtaggagtacgctgcatcgagatgctcattgagtcaac 3200
        S  K  G  L  L  G  G  E  A  W  S  S  W  H  K  E  D  S  I  H  V  G  V  R  C  I  E  M  L  I  E  S  T
     >>>>>>>>>>>>>>>>>>>>>>>>>>>>>>>>>>>>>>>>>>>>>>>>>>>>>>>>>>>>>>>>>>>>>>>>>>>>>>>>>>>>>>>>>>>>>>>>>
     I716104

              3210      3220      3230      3240      3250      3260      3270      3280      3290      3300
        *         *         *         *         *         *         *         *         *         *
3201 cggaatggttagcttacaccgccaaaatgctggcgtagtaggtcaagactctgagactatcgaactcgcacctgaatacgctgaggctatcgcaacccgt 3300
      G  M  V  S  L  H  R  Q  N  A  G  V  V  G  Q  D  S  E  T  I  E  L  A  P  E  Y  A  E  A  I  A  T  R
     >>>>>>>>>>>>>>>>>>>>>>>>>>>>>>>>>>>>>>>>>>>>>>>>>>>>>>>>>>>>>>>>>>>>>>>>>>>>>>>>>>>>>>>>>>>>>>>>>
     I716104

              3310      3320      3330      3340      3350      3360      3370      3380      3390      3400
        *         *         *         *         *         *         *         *         *         *
3301 gcaggtgcgctggctggcatctctccgatgttccaaccttgcgtagttcctcctaagccgtggactggcattactggtggtggctattgggctaacggtc 3400
     A  G  A  L  A  G  I  S  P  M  F  Q  P  C  V  V  P  P  K  P  W  T  G  I  T  G  G  G  Y  W  A  N  G  R
     >>>>>>>>>>>>>>>>>>>>>>>>>>>>>>>>>>>>>>>>>>>>>>>>>>>>>>>>>>>>>>>>>>>>>>>>>>>>>>>>>>>>>>>>>>>>>>>>>
     I716104

              3410      3420      3430      3440      3450      3460      3470      3480      3490      3500
        *         *         *         *         *         *         *         *         *         *
3401 gtcgtcctctggcgctggtgcgtactcacagtaagaaagcactgatgcgctacgaagacgtttacatgcctgaggtgtacaaagcgattaacattgcgca 3500
        R  P  L  A  L  V  R  T  H  S  K  K  A  L  M  R  Y  E  D  V  Y  M  P  E  V  Y  K  A  I  N  I  A  Q
     >>>>>>>>>>>>>>>>>>>>>>>>>>>>>>>>>>>>>>>>>>>>>>>>>>>>>>>>>>>>>>>>>>>>>>>>>>>>>>>>>>>>>>>>>>>>>>>>>
     I716104

              3510      3520      3530      3540      3550      3560      3570      3580      3590      3600
        *         *         *         *         *         *         *         *         *         *
3501 aaacaccgcatggaaaatcaacaagaaagtcctagcggtcgccaacgtaatcaccaagtggaagcattgtccggtcgaggacatccctgcgattgagcgt 3600
      N  T  A  W  K  I  N  K  K  V  L  A  V  A  N  V  I  T  K  W  K  H  C  P  V  E  D  I  P  A  I  E  R
     >>>>>>>>>>>>>>>>>>>>>>>>>>>>>>>>>>>>>>>>>>>>>>>>>>>>>>>>>>>>>>>>>>>>>>>>>>>>>>>>>>>>>>>>>>>>>>>>>
     I716104

              3610      3620      3630      3640      3650      3660      3670      3680      3690      3700
        *         *         *         *         *         *         *         *         *         *
3601 gaagaactcccgatgaaaccggaagacatcgacatgaatcctgaggctctcaccgcgtggaaacgtgctgccgctgctgtgtaccgcaaggacaaggctc 3700
     E  E  L  P  M  K  P  E  D  I  D  M  N  P  E  A  L  T  A  W  K  R  A  A  A  A  V  Y  R  K  D  K  A  R
     >>>>>>>>>>>>>>>>>>>>>>>>>>>>>>>>>>>>>>>>>>>>>>>>>>>>>>>>>>>>>>>>>>>>>>>>>>>>>>>>>>>>>>>>>>>>>>>>>
     I716104

              3710      3720      3730      3740      3750      3760      3770      3780      3790      3800
        *         *         *         *         *         *         *         *         *         *
3701 gcaagtctcgccgtatcagccttgagttcatgcttgagcaagccaataagtttgctaaccataaggccatctggttcccttacaacatggactggcgcgg 3800
        K  S  R  R  I  S  L  E  F  M  L  E  Q  A  N  K  F  A  N  H  K  A  I  W  F  P  Y  N  M  D  W  R  G
     >>>>>>>>>>>>>>>>>>>>>>>>>>>>>>>>>>>>>>>>>>>>>>>>>>>>>>>>>>>>>>>>>>>>>>>>>>>>>>>>>>>>>>>>>>>>>>>>>
     I716104

              3810      3820      3830      3840      3850      3860      3870      3880      3890      3900
        *         *         *         *         *         *         *         *         *         *
3801 tcgtgtttacgctgtgtcaatgttcaacccgcaaggtaacgatatgaccaaaggactgcttacgctggcgaaaggtaaaccaatcggtaaggaaggttac 3900
      R  V  Y  A  V  S  M  F  N  P  Q  G  N  D  M  T  K  G  L  L  T  L  A  K  G  K  P  I  G  K  E  G  Y
     >>>>>>>>>>>>>>>>>>>>>>>>>>>>>>>>>>>>>>>>>>>>>>>>>>>>>>>>>>>>>>>>>>>>>>>>>>>>>>>>>>>>>>>>>>>>>>>>>
     I716104

              3910      3920      3930      3940      3950      3960      3970      3980      3990      4000
        *         *         *         *         *         *         *         *         *         *
3901 tactggctgaaaatccacggtgcaaactgtgcgggtgtcgataaggttccgttccctgagcgcatcaagttcattgaggaaaaccacgagaacatcatgg 4000
     Y  W  L  K  I  H  G  A  N  C  A  G  V  D  K  V  P  F  P  E  R  I  K  F  I  E  E  N  H  E  N  I  M  A
     >>>>>>>>>>>>>>>>>>>>>>>>>>>>>>>>>>>>>>>>>>>>>>>>>>>>>>>>>>>>>>>>>>>>>>>>>>>>>>>>>>>>>>>>>>>>>>>>>
     I716104

              4010      4020      4030      4040      4050      4060      4070      4080      4090      4100
        *         *         *         *         *         *         *         *         *         *
4001 cttgcgctaagtctccactggagaacacttggtgggctgagcaagattctccgttctgcttccttgcgttctgctttgagtacgctggggtacagcacca 4100
        C  A  K  S  P  L  E  N  T  W  W  A  E  Q  D  S  P  F  C  F  L  A  F  C  F  E  Y  A  G  V  Q  H  H
     >>>>>>>>>>>>>>>>>>>>>>>>>>>>>>>>>>>>>>>>>>>>>>>>>>>>>>>>>>>>>>>>>>>>>>>>>>>>>>>>>>>>>>>>>>>>>>>>>
```

```
I716104

       4110      4120      4130      4140      4150      4160      4170      4180      4190      4200
    *         *         *         *         *         *         *         *         *         *
4101 cggcctgagctataactgctcccttccgctggcgtttgacgggtcttgctctggcatccagcacttctccgcgatgctccgagatgaggtaggtggtcgc 4200
      G  L  S  Y  N  C  S  L  P  L  A  F  D  G  S  C  S  G  I  Q  H  F  S  A  M  L  R  D  E  V  G  G  R
     >>>>>>>>>>>>>>>>>>>>>>>>>>>>>>>>>>>>>>>>>>>>>>>>>>>>>>>>>>>>>>>>>>>>>>>>>>>>>>>>>>>> >>>>>>>>>>
     I716104

       4210      4220      4230      4240      4250      4260      4270      4280      4290      4300
    *         *         *         *         *         *         *         *         *         *
4201 gcggttaacttgcttcctagtgaaaccgttcaggacatctacgggattgttgctaagaaagtcaacgagattctacaagcagacgcaatcaatgggaccg 4300
     A  V  N  L  L  P  S  E  T  V  Q  D  I  Y  G  I  V  A  K  K  V  N  E  I  L  Q  A  D  A  I  N  G  T  D
     >>>>>>>>>>>>>>>>>>>>>>>>>>>>>>>>>>>>>>>>>>>>>>>>>>>>>>>>>>>>>>>>>>>>>>>>>>>>>>>>>>>> >>>>>>>>>>
     I716104

       4310      4320      4330      4340      4350      4360      4370      4380      4390      4400
    *         *         *         *         *         *         *         *         *         *
4301 ataacgaagtagttaccgtgaccgatgagaacactggtgaaatctctgagaaagtcaagctgggcactaaggcactggctggtcaatggctggcttacgg 4400
       N  E  V  V  T  V  T  D  E  N  T  G  E  I  S  E  K  V  K  L  G  T  K  A  L  A  G  Q  W  L  A  Y  G
     >>>>>>>>>>>>>>>>>>>>>>>>>>>>>>>>>>>>>>>>>>>>>>>>>>>>>>>>>>>>>>>>>>>>>>>>>>>>>>>>>>>> >>>>>>>>>>
     I716104

       4410      4420      4430      4440      4450      4460      4470      4480      4490      4500
    *         *         *         *         *         *         *         *         *         *
4401 tgttactcgcagtgtgactaagcgttcagtcatgacgctggcttacgggtccaaagagttcggcttccgtcaacaagtgctggaagataccattcagcca 4500
      V  T  R  S  V  T  K  R  S  V  M  T  L  A  Y  G  S  K  E  F  G  F  R  Q  Q  V  L  E  D  T  I  Q  P
     >>>>>>>>>>>>>>>>>>>>>>>>>>>>>>>>>>>>>>>>>>>>>>>>>>>>>>>>>>>>>>>>>>>>>>>>>>>>>>>>>>>> >>>>>>>>>>
     I716104

       4510      4520      4530      4540      4550      4560      4570      4580      4590      4600
    *         *         *         *         *         *         *         *         *         *
4501 gctattgattccggcaagggtctgatgttcactcagccgaatcaggctgctggatacatggctaagctgatttgggaatctgtgagcgtgacggtggtag 4600
     A  I  D  S  G  K  G  L  M  F  T  Q  P  N  Q  A  A  G  Y  M  A  K  L  I  W  E  S  V  S  V  T  V  V  A
     >>>>>>>>>>>>>>>>>>>>>>>>>>>>>>>>>>>>>>>>>>>>>>>>>>>>>>>>>>>>>>>>>>>>>>>>>>>>>>>>>>>> >>>>>>>>>>
     I716104

       4610      4620      4630      4640      4650      4660      4670      4680      4690      4700
    *         *         *         *         *         *         *         *         *         *
4601 ctgcggttgaagcaatgaactggcttaagtctgctgctaagctgctggctgctgaggtcaaagataagaagactggagagattcttcgcaagcgttgcgc 4700
       A  V  E  A  M  N  W  L  K  S  A  A  K  L  L  A  A  E  V  K  D  K  K  T  G  E  I  L  R  K  R  C  A
     >>>>>>>>>>>>>>>>>>>>>>>>>>>>>>>>>>>>>>>>>>>>>>>>>>>>>>>>>>>>>>>>>>>>>>>>>>>>>>>>>>>> >>>>>>>>>>
     I716104

       4710      4720      4730      4740      4750      4760      4770      4780      4790      4800
    *         *         *         *         *         *         *         *         *         *
4701 tgtgcattgggtaactcctgatggtttccctgtgtggcaggaatacaagaagcctattcagacgcgcttgaacctgatgttcctcggtcagttccgctta 4800
      V  H  W  V  T  P  D  G  F  P  V  W  Q  E  Y  K  K  P  I  Q  T  R  L  N  L  M  F  L  G  Q  F  R  L
     >>>>>>>>>>>>>>>>>>>>>>>>>>>>>>>>>>>>>>>>>>>>>>>>>>>>>>>>>>>>>>>>>>>>>>>>>>>>>>>>>>>> >>>>>>>>>>
     I716104

       4810      4820      4830      4840      4850      4860      4870      4880      4890      4900
    *         *         *         *         *         *         *         *         *         *
4801 cagcctaccattaacaccaacaaagatagcgagattgatgcacacaaacaggagtctggtatcgctcctaactttgtacacagccaagacggtagccacc 4900
     Q  P  T  I  N  T  N  K  D  S  E  I  D  A  H  K  Q  E  S  G  I  A  P  N  F  V  H  S  Q  D  G  S  H  L
     >>>>>>>>>>>>>>>>>>>>>>>>>>>>>>>>>>>>>>>>>>>>>>>>>>>>>>>>>>>>>>>>>>>>>>>>>>>>>>>>>>>> >>>>>>>>>>
     I716104

       4910      4920      4930      4940      4950      4960      4970      4980      4990      5000
    *         *         *         *         *         *         *         *         *         *
4901 ttcgtaagactgtagtgtgggcacacgagaagtacggaatcgaatcttttgcactgattcacgactccttcggtaccattccggctgacgctgcgaacct 5000
       R  K  T  V  V  W  A  H  E  K  Y  G  I  E  S  F  A  L  I  H  D  S  F  G  T  I  P  A  D  A  A  N  L
     >>>>>>>>>>>>>>>>>>>>>>>>>>>>>>>>>>>>>>>>>>>>>>>>>>>>>>>>>>>>>>>>>>>>>>>>>>>>>>>>>>>> >>>>>>>>>>
     I716104

       5010      5020      5030      5040      5050      5060      5070      5080      5090      5100
```

```
         *         *         *         *         *         *         *         *         *         *
5001 gttcaaagcagtgcgcgaaactatggttgacacatatgagtcttgtgatgtactggctgatttctacgaccagttcgctgaccagttgcacgagtctcaa 5100
      F  K  A  V  R  E  T  M  V  D  T  Y  E  S  C  D  V  L  A  D  F  Y  D  Q  F  A  D  Q  L  H  E  S  Q
     >>>>>>>>>>>>>>>>>>>>>>>>>>>>>>>>>>>>>>>>>>>>>>>>>>>>>>>>>>>>>>>>>>>>>>>>>>>>>>>>>>>>>>>>>>>>>>>>>>>
     I716104

             5110      5120      5130      5140      5150      5160      5170      5180      5190      5200
         *         *         *         *         *         *         *         *         *         *
5101 ttggacaaaatgccagcacttccggctaaaggtaacttgaacctccgtgacatcttagagtcggacttcgcgttcgcgtaatactagtagcggccgctgc 5200
     L  D  K  M  P  A  L  P  A  K  G  N  L  N  L  R  D  I  L  E  S  D  F  A  F  A  *
     >>>>>>>>>>>>>>>>>>>>>>>>>>>>>>>>>>>>>>>>>>>>>>>>>>>>>>>>>>>>>>>>>>>>>>>>>>>>>>>>>
     I716104

             5210      5220      5230      5240      5250      5260      5270      5280      5290      5300
         *         *         *         *         *         *         *         *         *         *
5201 aggatatctggatccacgaagcttcccatggtgacgtcaccggttctagatacctaggtgagctctggtaccctctagtcaaggcctatagtgagtcgta 5300

             5310      5320      5330      5340      5350      5360      5370      5380      5390      5400
         *         *         *         *         *         *         *         *         *         *
5301 ttacggactggccgtcgttttacaacgtcgtgactgggaaaaccctggcgttacccaacttaatcgccttgcagcacatccccctttcgccagctggcgt 5400
                                                                                                 W  R
                                                                                                 >>>>>>
                                                                                                 LacZ alpha

             5410      5420      5430      5440      5450      5460      5470      5480      5490      5500
         *         *         *         *         *         *         *         *         *         *
5401 aatagcgaagaggcccgcaccgatcgcccttcccaacagttgcgcagcctgaatggcgaatggcgcttcgcttggtaataaagcccgcttcggcgggctt 5500
     N  S  E  E  A  R  T  D  R  P  S  Q  Q  L  R  S  L  N  G  E  W
     >>>>>>>>>>>>>>>>>>>>>>>>>>>>>>>>>>>>>>>>>>>>>>>>>>>>>>>>>>>>>>>
     LacZ alpha


         *
5501 tttttt 5506
```

Thu Jan 19, 2012 11:37 PST
J64997-B0034-E0051-pSB4C5.ape
Text Map

```
            10        20        30        40        50        60        70        80        90       100
     *         *         *         *         *         *         *         *         *         *
  1 taatacgactcactataggtagtagagaaagaggagaaatactagatgaccatgattacggattcactggccgtcgttttacaacgtcgtgactgggaaa 100
                                                  M  T  M  I  T  D  S  L  A  V  V  L  Q  R  R  D  W  E  N
                          >>>>>>>>>>>>           >>>>>>>>>>>>>>>>>>>>>>>>>>>>>>>>>>>>>>>>>>>>>>>>>>>>>
                          B0034                  E0051
    >>>>>>>>>>>>>>>>>>>
    J64997

           110       120       130       140       150       160       170       180       190       200
     *         *         *         *         *         *         *         *         *         *
101 accctggcgttacccaacttaatcgccttgcagcacatccccctttcgccagctggcgtaatagcgaagaggcccgcaccgatcgcccttcccaacagtt 200
      P  G  V  T  Q  L  N  R  L  A  A  H  P  P  F  A  S  W  R  N  S  E  E  A  R  T  D  R  P  S  Q  Q  L
    >>>>>>>>>>>>>>>>>>>>>>>>>>>>>>>>>>>>>>>>>>>>>>>>>>>>>>>>>>>>>>>>>>>>>>>>>>>>>>>>>>>>>>>>>>>>>>>>>>>>
    E0051

           210       220       230       240       250       260       270       280       290       300
     *         *         *         *         *         *         *         *         *         *
201 gcgcagcctgaatggcgaatggcgcgcgggcggcagcgaaggcggcggcagcgaacatcatcatcatcatcatggcagcgaacgtaaaggagaagaactt 300
     R  S  L  N  G  E  W  R  A  G  G  S  E  G  G  G  S  E  H  H  H  H  H  H  G  S  E  R  K  G  E  E  L
    >>>>>>>>>>>>>>>>>>>>>>>>>>>>>>>>>>>>>>>>>>>>>>>>>>>>>>>>>>>>>>>>>>>>>>>>>>>>>>>>>>>>>>>>>>>>>>>>>>>>
    E0051

           310       320       330       340       350       360       370       380       390       400
     *         *         *         *         *         *         *         *         *         *
301 ttcactggagttgtcccaattcttgttgaattagatggtgatgttaatgggcacaaattttctgtcagtggagagggtgaaggtgatgcaacatacggaa 400
    F  T  G  V  V  P  I  L  V  E  L  D  G  D  V  N  G  H  K  F  S  V  S  G  E  G  E  G  D  A  T  Y  G  K
    >>>>>>>>>>>>>>>>>>>>>>>>>>>>>>>>>>>>>>>>>>>>>>>>>>>>>>>>>>>>>>>>>>>>>>>>>>>>>>>>>>>>>>>>>>>>>>>>>>>>
    E0051

           410       420       430       440       450       460       470       480       490       500
     *         *         *         *         *         *         *         *         *         *
401 aacttacccttaaatttatttgcactactggaaaactacctgttccatggccaacacttgtcactaccctgacctatggtgttcaatgctttgcgagata 500
      L  T  L  K  F  I  C  T  T  G  K  L  P  V  P  W  P  T  L  V  T  T  L  T  Y  G  V  Q  C  F  A  R  Y
    >>>>>>>>>>>>>>>>>>>>>>>>>>>>>>>>>>>>>>>>>>>>>>>>>>>>>>>>>>>>>>>>>>>>>>>>>>>>>>>>>>>>>>>>>>>>>>>>>>>>
    E0051

           510       520       530       540       550       560       570       580       590       600
     *         *         *         *         *         *         *         *         *         *
501 cccagatcatatgaaacagcatgactttttcaagagtgccatgcccgaaggttatgtacaggaaagaactatatttttcaaagatgacgggaactacaag 600
     P  D  H  M  K  Q  H  D  F  F  K  S  A  M  P  E  G  Y  V  Q  E  R  T  I  F  F  K  D  D  G  N  Y  K
    >>>>>>>>>>>>>>>>>>>>>>>>>>>>>>>>>>>>>>>>>>>>>>>>>>>>>>>>>>>>>>>>>>>>>>>>>>>>>>>>>>>>>>>>>>>>>>>>>>>>
    E0051

           610       620       630       640       650       660       670       680       690       700
     *         *         *         *         *         *         *         *         *         *
601 acacgtgctgaagtcaagtttgaaggtgatacccttgttaatagaatcgagttaaaaggtattgattttaaagaagatggaaacattcttggacacaaat 700
    T  R  A  E  V  K  F  E  G  D  T  L  V  N  R  I  E  L  K  G  I  D  F  K  E  D  G  N  I  L  G  H  K  L
    >>>>>>>>>>>>>>>>>>>>>>>>>>>>>>>>>>>>>>>>>>>>>>>>>>>>>>>>>>>>>>>>>>>>>>>>>>>>>>>>>>>>>>>>>>>>>>>>>>>>
    E0051

           710       720       730       740       750       760       770       780       790       800
     *         *         *         *         *         *         *         *         *         *
701 tggaatacaactataactcacacaatgtatacatcatggcagacaaacaaaagaatggaatcaaagttaacttcaaaattagacacaacattgaagatgg 800
      E  Y  N  Y  N  S  H  N  V  Y  I  M  A  D  K  Q  K  N  G  I  K  V  N  F  K  I  R  H  N  I  E  D  G
    >>>>>>>>>>>>>>>>>>>>>>>>>>>>>>>>>>>>>>>>>>>>>>>>>>>>>>>>>>>>>>>>>>>>>>>>>>>>>>>>>>>>>>>>>>>>>>>>>>>>
    E0051

           810       820       830       840       850       860       870       880       890       900
     *         *         *         *         *         *         *         *         *         *
801 aagcgttcaactagcagaccattatcaacaaaatactccaattggcgatggccctgtccttttaccagacaaccattacctgtccacacaatctgccctt 900
     S  V  Q  L  A  D  H  Y  Q  Q  N  T  P  I  G  D  G  P  V  L  L  P  D  N  H  Y  L  S  T  Q  S  A  L
    >>>>>>>>>>>>>>>>>>>>>>>>>>>>>>>>>>>>>>>>>>>>>>>>>>>>>>>>>>>>>>>>>>>>>>>>>>>>>>>>>>>>>>>>>>>>>>>>>>>>
```

```
E0051

        910       920       930       940       950       960       970       980       990       1000
          *         *         *         *         *         *         *         *         *         *
 901 tcgaaagatcccaacgaaaagagagaccacatggtccttcttgagtttgtaacagctgctgggattacacatggcatggatgaactatacaaataataat 1000
     S  K  D  P  N  E  K  R  D  H  M  V  L  L  E  F  V  T  A  A  G  I  T  H  G  M  D  E  L  Y  K  *  *
     >>>>>>>>>>>>>>>>>>>>>>>>>>>>>>>>>>>>>>>>>>>>>>>>>>>>>>>>>>>>>>>>>>>>>>>>>>>>>>>>>>>>>>>>>>>>>>>>>>>
     E0051

       1010      1020      1030      1040      1050      1060      1070      1080      1090      1100
          *         *         *         *         *         *         *         *         *         *
1001 actagtagcggccgctgcaggagtcactaagggttagttagttagattagcagaaagtcaaaagcctccgaccggaggcttttgactaaaacttcccttg 1100

       1110      1120      1130      1140      1150      1160      1170      1180      1190      1200
          *         *         *         *         *         *         *         *         *         *
1101 gggttatcattggggctcactcaaaggcggtaatcagataaaaaaaatccttagctttcgctaaggatgatttctgctagagctgtcagaccaagtttac 1200

       1210      1220      1230      1240      1250      1260      1270      1280      1290      1300
          *         *         *         *         *         *         *         *         *         *
1201 gagctcgcttggactcctgttgatagatccagtaatgacctcagaactccatctggatttgttcagaacgctcggttgccgccgggcgtttttttattggt 1300

       1310      1320      1330      1340      1350      1360      1370      1380      1390      1400
          *         *         *         *         *         *         *         *         *         *
1301 gagaatccaagcactagggacagtaagacgggtaagcctgttgatgataccgctgccttactgggtgcattagccagtctgaatgacctgtcacgggata 1400

       1410      1420      1430      1440      1450      1460      1470      1480      1490      1500
          *         *         *         *         *         *         *         *         *         *
1401 atccgaagtggtcagactggaaaatcagagggcaggaactgctgaacagcaaaaagtcagatagcaccacatagcagacccgccataaaacgccctgaga 1500

       1510      1520      1530      1540      1550      1560      1570      1580      1590      1600
          *         *         *         *         *         *         *         *         *         *
1501 agcccgtgacgggcttttcttgtattatgggtagtttccttgcatgaatccataaaaggcgcctgtagtgccatttacccccattcactgccagagccgt 1600

       1610      1620      1630      1640      1650      1660      1670      1680      1690      1700
          *         *         *         *         *         *         *         *         *         *
1601 gagcgcagcgaactgaatgtcacgaaaaagacagcgactcaggtgcctgatggtcggagacaaaaggaatattcagcgatttgcccgagcttgcgagggt 1700

       1710      1720      1730      1740      1750      1760      1770      1780      1790      1800
          *         *         *         *         *         *         *         *         *         *
1701 gctacttaagcctttagggttttaaggtctgttttgtagaggagcaaacagcgtttgcgacatccttttgtaatactgcggaactgactaaagtagtgag 1800

       1810      1820      1830      1840      1850      1860      1870      1880      1890      1900
          *         *         *         *         *         *         *         *         *         *
1801 ttatacacagggctgggatctattctttttatctttttttattctttctttattctataaattataaccacttgaatataaacaaaaaaaacacacaaag 1900

       1910      1920      1930      1940      1950      1960      1970      1980      1990      2000
          *         *         *         *         *         *         *         *         *         *
1901 gtctagcggaatttacagagggtctagcagaatttacaagttttccagcaaaggtctagcagaatttacagatacccacaactcaaaggaaaaggacatg 2000

       2010      2020      2030      2040      2050      2060      2070      2080      2090      2100
          *         *         *         *         *         *         *         *         *         *
2001 taattatcattgactagcccatctcaattggtatagtgattaaaatcacctagaccaattgagatgtatgtctgaattagttgttttcaaagcaaatgaa 2100

       2110      2120      2130      2140      2150      2160      2170      2180      2190      2200
          *         *         *         *         *         *         *         *         *         *
2101 ctagcgattagtcgctatgacttaacggagcatgaaaccaagctaattttatgctgtgtggcactactcaaccccacgattgaaaaccctacaaggaaag 2200

       2210      2220      2230      2240      2250      2260      2270      2280      2290      2300
          *         *         *         *         *         *         *         *         *         *
2201 aacggacggtatcgttcacttataaccaatacgctcagatgatgaacatcagtagggaaaatgcttatggtgtattagctaaagcaaccagagagctgat 2300

       2310      2320      2330      2340      2350      2360      2370      2380      2390      2400
          *         *         *         *         *         *         *         *         *         *
2301 gacgagaactgtggaaatcaggaatcctttggttaaaggctttgagattttccagtggacaaactatgccaagttctcaagcgaaaaattagaattagtt 2400

       2410      2420      2430      2440      2450      2460      2470      2480      2490      2500
```

```
          *         *         *         *         *         *         *         *         *         *
2401 tttagtgaagagatattgccttatcttttccagttaaaaaaattcataaaatataatctggaacatgttaagtcttttgaaaacaaatactctatgagga 2500

          2510      2520      2530      2540      2550      2560      2570      2580      2590      2600
          *         *         *         *         *         *         *         *         *         *
2501 tttatgagtggttattaaaagaactaacacaaaagaaaactcacaaggcaaatatagagattagccttgatgaatttaagttcatgttaatgcttgaaaa 2600

          2610      2620      2630      2640      2650      2660      2670      2680      2690      2700
          *         *         *         *         *         *         *         *         *         *
2601 taactaccatgagtttaaaaggcttaaccaatgggttttgaaaccaataagtaaagatttaaacacttacagcaatatgaaattggtggttgataagcga 2700

          2710      2720      2730      2740      2750      2760      2770      2780      2790      2800
          *         *         *         *         *         *         *         *         *         *
2701 ggccgcccgactgatacgttgattttccaagttgaactagatagacaaatggatctcgtaaccgaacttgagaacaaccagataaaaatgaatggtgaca 2800

          2810      2820      2830      2840      2850      2860      2870      2880      2890      2900
          *         *         *         *         *         *         *         *         *         *
2801 aaataccaacaaccattacatcagattcctacctacgtaacggactaagaaaaacactacacgatgctttaactgcaaaaattcagctcaccagttttga 2900

          2910      2920      2930      2940      2950      2960      2970      2980      2990      3000
          *         *         *         *         *         *         *         *         *         *
2901 ggcaaaattttgagtgacatgcaaagtaagcatgatctcaatggttcgttctcatggctcacgcaaaaacaacgaaccacactagagaacatactggct 3000

          3010      3020      3030      3040      3050      3060      3070      3080      3090      3100
          *         *         *         *         *         *         *         *         *         *
3001 aaatacggaaggatctgaggttcttatggctcttgtatctatcagtgaagcatcaagactaacaaacaaaagtagaacaactgttcaccgttagatatca 3100

          3110      3120      3130      3140      3150      3160      3170      3180      3190      3200
          *         *         *         *         *         *         *         *         *         *
3101 aagggaaaactgtccatatgcacagatgaaaacggtgtaaaaaagatagatacatcagagcttttacgagtttttggtgcatttaaagctgttcaccatg 3200

          3210      3220      3230      3240      3250      3260      3270      3280      3290      3300
          *         *         *         *         *         *         *         *         *         *
3201 aacagatcgacaatgtaactactagaggttgatcgggcacgtaagaggttccaactttcaccataatgaaataagatcactaccgggcgtatttttttgag 3300

          3310      3320      3330      3340      3350      3360      3370      3380      3390      3400
          *         *         *         *         *         *         *         *         *         *
3301 ttatcgagattttcaggagctaaggaagctaaaatggagaaaaaaatcacgggatataccaccgttgatatatcccaatggcatcgtaaagaacattttg 3400

          3410      3420      3430      3440      3450      3460      3470      3480      3490      3500
          *         *         *         *         *         *         *         *         *         *
3401 aggcatttcagtcagttgctcaatgtacctataaccagaccgttcagctggatattacggcctttttaaagaccgtaaagaaaaataagcacaagtttta 3500

          3510      3520      3530      3540      3550      3560      3570      3580      3590      3600
          *         *         *         *         *         *         *         *         *         *
3501 tccggcctttattcacattcttgcccgcctgatgaacgctcacccggagtttcgtatggccatgaaagacggtgagctggtgatctgggatagtgttcac 3600

          3610      3620      3630      3640      3650      3660      3670      3680      3690      3700
          *         *         *         *         *         *         *         *         *         *
3601 ccttgttacaccgttttccatgagcaaactgaaacgttttcgtccctctggagtgaataccacgacgatttccggcagtttctccacatatattcgcaag 3700

          3710      3720      3730      3740      3750      3760      3770      3780      3790      3800
          *         *         *         *         *         *         *         *         *         *
3701 atgtggcgtgttacggtgaaaacctggcctatttccctaaagggtttattgagaatatgtttttgtctcagccaatccctgggtgagtttcaccagttt 3800

          3810      3820      3830      3840      3850      3860      3870      3880      3890      3900
          *         *         *         *         *         *         *         *         *         *
3801 tgatttaaacgtggccaatatggacaacttcttcgcccccgttttcacgatgggcaaatattatacgcaaggcgacaaggtgctgatgccgctggcgatc 3900

          3910      3920      3930      3940      3950      3960      3970      3980      3990      4000
          *         *         *         *         *         *         *         *         *         *
3901 caggttcatcatgccgtttgtgatggcttccatgtcggccgcatgcttaatgaattacaacagtactgtgatgagtggcagggcggggcgtaataatact 4000

          4010      4020      4030      4040      4050      4060      4070      4080      4090      4100
          *         *         *         *         *         *         *         *         *         *
4001 agctccggcaaaaaaacgggcaaggtgtcaccaccctgccctttttctttaaaaccgaaaagattacttcgcgtttgccacctgacgtctaagaaaagga 4100
```

```
           4110      4120      4130      4140      4150      4160      4170      4180      4190      4200
        *         *         *         *         *         *         *         *         *         *
4101 atattcagcaatttgcccgtgccgaagaaaggcccacccgtgaaggtgagccagtgagttgattgctacgtaattagttagttagcccttagtgactcga 4200

           4210      4220
        *         *
4201 attcgcggccgcttctagag 4220
```

Thu Jan 19, 2012 11:40 PST
J23115-B0032-GFP-Litmus28i.ape
Text Map

```
                10        20        30        40        50        60        70        80        90       100
           *         *         *         *         *         *         *         *         *         *
     1 gttaactacgtcaggtggcacttttcggggaaatgtgcgcggaacccctatttgtttatttttctaaatacattcaaatatgtatccgctcatgagacaa 100

                110       120       130       140       150       160       170       180       190       200
           *         *         *         *         *         *         *         *         *         *
   101 taaccctgataaatgcttcaataatattgaaaaaggaagagtatgagtattcaacatttccgtgtcgcccttattcccttttttgcggcattttgccttc 200

                210       220       230       240       250       260       270       280       290       300
           *         *         *         *         *         *         *         *         *         *
   201 ctgtttttgctcacccagaaacgctggtgaaagtaaaagatgctgaagatcagttgggtgcacgagtgggttacatcgaactggatctcaacagcggtaa 300

                310       320       330       340       350       360       370       380       390       400
           *         *         *         *         *         *         *         *         *         *
   301 gatccttgagagttttcgccccgaagaacgttctccaatgatgagcacttttaaagttctgctatgtggcgcggtattatcccgtgttgacgccgggcaa 400
                                               M  S  T  F  K  V  L  L  C  G  A  V  L  S  R  V  D  A  G  Q
                                               >>>>>>>>>>>>>>>>>>>>>>>>>>>>>>>>>>>>>>>>>>>>>>>>>>>>>>>>>>
                                               AmpR

                410       420       430       440       450       460       470       480       490       500
           *         *         *         *         *         *         *         *         *         *
   401 gagcaactcggtcgccgcatacactattctcagaatgacttggttgagtactcaccagtcacagaaaagcatcttacggatggcatgacagtaagagaat 500
       E  Q  L  G  R  R  I  H  Y  S  Q  N  D  L  V  E  Y  S  P  V  T  E  K  H  L  T  D  G  M  T  V  R  E  L
       >>>>>>>>>>>>>>>>>>>>>>>>>>>>>>>>>>>>>>>>>>>>>>>>>>>>>>>>>>>>>>>>>>>>>>>>>>>>>>>>>>>>>>>>>>>>>>>>>
       AmpR

                510       520       530       540       550       560       570       580       590       600
           *         *         *         *         *         *         *         *         *         *
   501 tatgcagtgctgccataaccatgagtgataacactgcggccaacttacttctgacaacgatcggaggaccgaaggagctaaccgcttttttgcacaacat 600
         C  S  A  A  I  T  M  S  D  N  T  A  A  N  L  L  L  T  T  I  G  G  P  K  E  L  T  A  F  L  H  N  M
       >>>>>>>>>>>>>>>>>>>>>>>>>>>>>>>>>>>>>>>>>>>>>>>>>>>>>>>>>>>>>>>>>>>>>>>>>>>>>>>>>>>>>>>>>>>>>>>>>
       AmpR

                610       620       630       640       650       660       670       680       690       700
           *         *         *         *         *         *         *         *         *         *
   601 ggggggatcatgtaactcgccttgatcgttgggaaccggagctgaatgaagccataccaaacgacgagcgtgacaccacgatgcctgtagcaatggcaaca 700
        G  D  H  V  T  R  L  D  R  W  E  P  E  L  N  E  A  I  P  N  D  E  R  D  T  T  M  P  V  A  M  A  T
       >>>>>>>>>>>>>>>>>>>>>>>>>>>>>>>>>>>>>>>>>>>>>>>>>>>>>>>>>>>>>>>>>>>>>>>>>>>>>>>>>>>>>>>>>>>>>>>>>
       AmpR

                710       720       730       740       750       760       770       780       790       800
           *         *         *         *         *         *         *         *         *         *
   701 acgttgcgcaaactattaactggcgaactacttactctagcttcccggcaacaattaatagactggatggaggcggataaagttgcaggaccacttctgc 800
       T  L  R  K  L  L  T  G  E  L  L  T  L  A  S  R  Q  Q  L  I  D  W  M  E  A  D  K  V  A  G  P  L  L  R
       >>>>>>>>>>>>>>>>>>>>>>>>>>>>>>>>>>>>>>>>>>>>>>>>>>>>>>>>>>>>>>>>>>>>>>>>>>>>>>>>>>>>>>>>>>>>>>>>>
       AmpR

                810       820       830       840       850       860       870       880       890       900
           *         *         *         *         *         *         *         *         *         *
   801 gctcggcccttccggctggctggtttattgctgataaatctggagccggtgagcgtgggtctcgcggtatcattgcagcactggggccagatggtaagcc 900
         S  A  L  P  A  G  W  F  I  A  D  K  S  G  A  G  E  R  G  S  R  G  I  I  A  A  L  G  P  D  G  K  P
       >>>>>>>>>>>>>>>>>>>>>>>>>>>>>>>>>>>>>>>>>>>>>>>>>>>>>>>>>>>>>>>>>>>>>>>>>>>>>>>>>>>>>>>>>>>>>>>>>
       AmpR

                910       920       930       940       950       960       970       980       990      1000
           *         *         *         *         *         *         *         *         *         *
   901 ctcccgtatcgtagttatctacacgacggggagtcaggcaactatggatgaacgaaatagacagatcgctgagataggtgcctcactgattaagcattgg 1000
        S  R  I  V  V  I  Y  T  T  G  S  Q  A  T  M  D  E  R  N  R  Q  I  A  E  I  G  A  S  L  I  K  H  W
       >>>>>>>>>>>>>>>>>>>>>>>>>>>>>>>>>>>>>>>>>>>>>>>>>>>>>>>>>>>>>>>>>>>>>>>>>>>>>>>>>>>>>>>>>>>>>>>>>
       AmpR

               1010      1020      1030      1040      1050      1060      1070      1080      1090      1100
           *         *         *         *         *         *         *         *         *         *
```

```
1001 taactgtcagaccaagtttactcatatatactttagattgatttaccccggttgataatcagaaaagccccaaaaacaggaagattgtataagcaaatat 1100

              1110      1120      1130      1140      1150      1160      1170      1180      1190      1200
        *         *         *         *         *         *         *         *         *         *
1101 ttaaattgtaaacgttaatattttgttaaaattcgcgttaaatttttgttaaatcagctcattttttaaccaataggccgaaatcggcaaaatcccttat 1200
       <<<<<<<<<<<<<<<<<<<<<<<<<<<<<<<<<<<<<<<<<<<<<<<<<<<<<<<<<<<<<<<<<<<<<<<<<<<<<<<<<<<<<<<<<<<<<<<<<<
       M13 origin

              1210      1220      1230      1240      1250      1260      1270      1280      1290      1300
        *         *         *         *         *         *         *         *         *         *
1201 aaatcaaaagaatagcccgagatagggttgagtgttgttccagtttggaacaagagtccactattaaagaacgtggactccaacgtcaaagggcgaaaaa 1300
     <<<<<<<<<<<<<<<<<<<<<<<<<<<<<<<<<<<<<<<<<<<<<<<<<<<<<<<<<<<<<<<<<<<<<<<<<<<<<<<<<<<<<<<<<<<<<<<<<<<<
     M13 origin

              1310      1320      1330      1340      1350      1360      1370      1380      1390      1400
        *         *         *         *         *         *         *         *         *         *
1301 ccgtctatcagggcgatggcccactacgtgaaccatcacccaaatcaagttttttggggtcgaggtgccgtaaagcactaaatcggaaccctaaagggag 1400
     <<<<<<<<<<<<<<<<<<<<<<<<<<<<<<<<<<<<<<<<<<<<<<<<<<<<<<<<<<<<<<<<<<<<<<<<<<<<<<<<<<<<<<<<<<<<<<<<<<<<
     M13 origin

              1410      1420      1430      1440      1450      1460      1470      1480      1490      1500
        *         *         *         *         *         *         *         *         *         *
1401 cccccgatttagagcttgacggggaaagcgaacgtggcgagaaaggaagggaagaaagcgaaaggagcgggcgctagggcgctggcaagtgtagcggtca 1500
     <<<<<<<<<<<<<<<<<<<<<<<<<<<<<<<<<<<<<<<<<<<<<<<<<<<<<<<<<<<<<<<<<<<<<<<<<<<<<<<<<<<<<<<<<<<<<<<<<<<<
     M13 origin

              1510      1520      1530      1540      1550      1560      1570      1580      1590      1600
        *         *         *         *         *         *         *         *         *         *
1501 cgctgcgcgtaaccaccacacccgccgcgcttaatgcgccgctacagggcgcgtaaaaggatctaggtgaagatcctttttgataatctcatgaccaaaa 1600
                                                                                             >>>>>>>>>>>>
                                                                                             ColE1 origin

     <<<<<<<<<<<<<<<<<<<<<<<<<<<<<<<<<<<<<<<<<<<<<<<<<<<<<<
     M13 origin

              1610      1620      1630      1640      1650      1660      1670      1680      1690      1700
        *         *         *         *         *         *         *         *         *         *
1601 tcccttaacgtgagttttcgttccactgagcgtcagaccccgtagaaaagatcaaaggatcttcttgagatcctttttttctgcgcgtaatctgctgctt 1700
     >>>>>>>>>>>>>>>>>>>>>>>>>>>>>>>>>>>>>>>>>>>>>>>>>>>>>>>>>>>>>>>>>>>>>>>>>>>>>>>>>>>>>>>>>>>>>>>>>>>>
     ColE1 origin

              1710      1720      1730      1740      1750      1760      1770      1780      1790      1800
        *         *         *         *         *         *         *         *         *         *
1701 gcaaacaaaaaaaccaccgctaccagcggtggtttgtttgccggatcaagagctaccaactctttttccgaaggtaactggcttcagcagagcgcagata 1800
     >>>>>>>>>>>>>>>>>>>>>>>>>>>>>>>>>>>>>>>>>>>>>>>>>>>>>>>>>>>>>>>>>>>>>>>>>>>>>>>>>>>>>>>>>>>>>>>>>>>>
     ColE1 origin

              1810      1820      1830      1840      1850      1860      1870      1880      1890      1900
        *         *         *         *         *         *         *         *         *         *
1801 ccaaatactgttcttctagtgtagccgtagttaggccaccacttcaagaactctgtagcaccgcctacatacctcgctctgctaatcctgttaccagtgg 1900
     >>>>>>>>>>>>>>>>>>>>>>>>>>>>>>>>>>>>>>>>>>>>>>>>>>>>>>>>>>>>>>>>>>>>>>>>>>>>>>>>>>>>>>>>>>>>>>>>>>>>
     ColE1 origin

              1910      1920      1930      1940      1950      1960      1970      1980      1990      2000
        *         *         *         *         *         *         *         *         *         *
1901 ctgctgccagtggcgataagtcgtgtcttaccgggttggactcaagacgatagttaccggataaggcgcagcggtcgggctgaacggggggttcgtgcac 2000
     >>>>>>>>>>>>>>>>>>>>>>>>>>>>>>>>>>>>>>>>>>>>>>>>>>>>>>>>>>>>>>>>>>>>>>>>>>>>>>>>>>>>>>>>>>>>>>>>>>>>
     ColE1 origin

              2010      2020      2030      2040      2050      2060      2070      2080      2090      2100
        *         *         *         *         *         *         *         *         *         *
2001 acagcccagcttggagcgaacgacctacaccgaactgagatacctacagcgtgagctatgagaaagcgccacgcttcccgaagggagaaaggcggacagg 2100
     >>>>>>>>>>>>>>>>>>>>>>>>>>>>>>>>>>>>>>>>>>>>>>>>>>>>>>>>>>>>>>>>>>>>>>>>>>>>>>>>>>>>>>>>>>>>>>>>>>>>
     ColE1 origin

              2110      2120      2130      2140      2150      2160      2170      2180      2190      2200
        *         *         *         *         *         *         *         *         *         *
```

```
2101 tatccggtaagcggcagggtcggaacaggagagcgcacgagggagcttccagggggaaacgcctggtatctttatagtcctgtcgggtttcgccacctct 2200
     >>>>>>>>>>>>>>>>>>>>>>>>>>>>>>>>>>>>>>>>>>>>>>>>>>>>>>>>>>>>>>>>>>>>>>>>>>>>>>>>>>>>>>>>>>>>>>>>>>>
     ColE1 origin

           2210      2220      2230      2240      2250      2260      2270      2280      2290      2300
         *         *         *         *         *         *         *         *         *         *
2201 gacttgagcgtcgatttttgtgatgctcgtcaggggggcggagcctatggaaaaacgccagcaacgcggcctttttacggttcctggccttttgctggcc 2300
     >>>>>>>>>>>>>>>>>>>>>>>>>>>>>>>>>>>>>>>>>>>>>>>>>>>>>>>>>>>>>>>>>>>>>>>
     ColE1 origin

           2310      2320      2330      2340      2350      2360      2370      2380      2390      2400
         *         *         *         *         *         *         *         *         *         *
2301 ttttgctcacatgtaatgtgagttagctcactcattaggcaccccaggctttacactttatgcttccggctcgtatgttgtgtggaattgtgagcggata 2400
                                                                                        >>>>>>>>>>>>>>>>>
                                                                                        LacO

           2410      2420      2430      2440      2450      2460      2470      2480      2490      2500
         *         *         *         *         *         *         *         *         *         *
2401 acaatttcacacaggaaacagctatgaccatgattacgccaagctacgtaatacgactcactatagggcagatcttcgaatgcatcgcgcgcaccgtacg 2500
                                                     >>>>>>>>>>>>>>>>>>>>>
                                                     T7

     >>>>>>
     LacO

           2510      2520      2530      2540      2550      2560      2570      2580      2590      2600
         *         *         *         *         *         *         *         *         *         *
2501 tctcgaggaattcgcggccgcttctagagtttatagctagctcagcccttggtacaatgctagctagtagagtcacacaggaaagtagtagatgcgtaaa 2600
                                                                                                M  R  K
                                   >>>>>>>>>>>>>>>>>>>>>>>>>>>>>>>>>>>        >>>>>>>>>>>>>      >>>>>>>>>
                                   J23115                                     B0032              GFP

           2610      2620      2630      2640      2650      2660      2670      2680      2690      2700
         *         *         *         *         *         *         *         *         *         *
2601 ggagaagaacttttcactggagttgtcccaattcttgttgaattagatggtgatgttaatgggcacaaattttctgtcagtggagagggtgaaggtgatg 2700
     G  E  E  L  F  T  G  V  V  P  I  L  V  E  L  D  G  D  V  N  G  H  K  F  S  V  S  G  E  G  E  G  D  A
     >>>>>>>>>>>>>>>>>>>>>>>>>>>>>>>>>>>>>>>>>>>>>>>>>>>>>>>>>>>>>>>>>>>>>>>>>>>>>>>>>>>>>>>>>>>>>>>>>>>
     GFP

           2710      2720      2730      2740      2750      2760      2770      2780      2790      2800
         *         *         *         *         *         *         *         *         *         *
2701 caacatacggaaaacttacccttaaatttatttgcactactggaaaactacctgttccatggccaacacttgtcactactttcggttatggtgttcaatg 2800
       T  Y  G  K  L  T  L  K  F  I  C  T  T  G  K  L  P  V  P  W  P  T  L  V  T  T  F  G  Y  G  V  Q  C
     >>>>>>>>>>>>>>>>>>>>>>>>>>>>>>>>>>>>>>>>>>>>>>>>>>>>>>>>>>>>>>>>>>>>>>>>>>>>>>>>>>>>>>>>>>>>>>>>>>>
     GFP

           2810      2820      2830      2840      2850      2860      2870      2880      2890      2900
         *         *         *         *         *         *         *         *         *         *
2801 ctttgcgagatacccagatcatatgaaacagcatgactttttcaagagtgccatgcccgaaggttatgtacaggaaagaactatatttttcaaagatgac 2900
      F  A  R  Y  P  D  H  M  K  Q  H  D  F  F  K  S  A  M  P  E  G  Y  V  Q  E  R  T  I  F  F  K  D  D
     >>>>>>>>>>>>>>>>>>>>>>>>>>>>>>>>>>>>>>>>>>>>>>>>>>>>>>>>>>>>>>>>>>>>>>>>>>>>>>>>>>>>>>>>>>>>>>>>>>>
     GFP

           2910      2920      2930      2940      2950      2960      2970      2980      2990      3000
         *         *         *         *         *         *         *         *         *         *
2901 gggaactacaagacacgtgctgaagtcaagtttgaaggtgatacccttgttaatagaatcgagttaaaaggtattgattttaaagaagatggaaacattc 3000
     G  N  Y  K  T  R  A  E  V  K  F  E  G  D  T  L  V  N  R  I  E  L  K  G  I  D  F  K  E  D  G  N  I  L
     >>>>>>>>>>>>>>>>>>>>>>>>>>>>>>>>>>>>>>>>>>>>>>>>>>>>>>>>>>>>>>>>>>>>>>>>>>>>>>>>>>>>>>>>>>>>>>>>>>>
     GFP

           3010      3020      3030      3040      3050      3060      3070      3080      3090      3100
         *         *         *         *         *         *         *         *         *         *
3001 ttggacacaaattggaatacaactataactcacacaatgtatacatcatggcagacaaacaaaagaatggaatcaaagttaacttcaaaattagacacaa 3100
       G  H  K  L  E  Y  N  Y  N  S  H  N  V  Y  I  M  A  D  K  Q  K  N  G  I  K  V  N  F  K  I  R  H  N
     >>>>>>>>>>>>>>>>>>>>>>>>>>>>>>>>>>>>>>>>>>>>>>>>>>>>>>>>>>>>>>>>>>>>>>>>>>>>>>>>>>>>>>>>>>>>>>>>>>>
     GFP
```

```
           3110      3120      3130      3140      3150      3160      3170      3180      3190      3200
         *         *         *         *         *         *         *         *         *         *
3101 cattgaagatggaagcgttcaactagcagaccattatcaacaaaatactccaattggcgatggccctgtccttttaccagacaaccattacctgtccaca 3200
      I  E  D  G  S  V  Q  L  A  D  H  Y  Q  Q  N  T  P  I  G  D  G  P  V  L  L  P  D  N  H  Y  L  S  T
     >>>>>>>>>>>>>>>>>>>>>>>>>>>>>>>>>>>>>>>>>>>>>>>>>>>>>>>>>>>>>>>>>>>>>>>>>>>>>>>>>>>>>>>>>>>>>>>>>>>>
     GFP

           3210      3220      3230      3240      3250      3260      3270      3280      3290      3300
         *         *         *         *         *         *         *         *         *         *
3201 caatctgccctttcgaaagatcccaacgaaaagagagaccacatggtccttcttgagtttgtaacagctgctgggattacacatggcatggatgaactat 3300
     Q  S  A  L  S  K  D  P  N  E  K  R  D  H  M  V  L  L  E  F  V  T  A  A  G  I  T  H  G  M  D  E  L  Y
     >>>>>>>>>>>>>>>>>>>>>>>>>>>>>>>>>>>>>>>>>>>>>>>>>>>>>>>>>>>>>>>>>>>>>>>>>>>>>>>>>>>>>>>>>>>>>>>>>>>>
     GFP

           3310      3320      3330      3340      3350      3360      3370      3380      3390      3400
         *         *         *         *         *         *         *         *         *         *
3301 acaaataataatactagtagcggccgctgcaggatatctggatccacgaagcttcccatggtgacgtcaccggttctagatacctaggtgagctctggta 3400
       K  *  *
     >>>>>>>>>>>
     GFP

           3410      3420      3430      3440      3450      3460      3470      3480      3490      3500
         *         *         *         *         *         *         *         *         *         *
3401 ccctctagtcaaggcctatagtgagtcgtattacggactggccgtcgttttacaacgtcgtgactgggaaaaccctggcgttacccaacttaatcgcctt 3500

           3510      3520      3530      3540      3550      3560      3570      3580      3590      3600
         *         *         *         *         *         *         *         *         *         *
3501 gcagcacatcccccttttcgccagctggcgtaatagcgaagaggcccgcaccgatcgcccttcccaacagttgcgcagcctgaatggcgaatggcgcttcg 3600
                             W  R  N  S  E  E  A  R  T  D  R  P  S  Q  Q  L  R  S  L  N  G  E  W
                             >>>>>>>>>>>>>>>>>>>>>>>>>>>>>>>>>>>>>>>>>>>>>>>>>>>>>>>>>>>>>>>>>>>>>>
                             LacZ alpha

           3610      3620      3630
         *         *         *         *
3601 cttggtaataaagcccgcttcggcgggcttttttt 3636
```

Thu Jan 19, 2012 11:38 PST
J23119-RBSCDog-mKate2-pSB4C5.ape
Text Map

```
            10        20        30        40        50        60        70        80        90       100
        *         *         *         *         *         *         *         *         *         *
  1 gaattcgcggccgcttctagagTTGACAGCTAGCTCAGTCCTAGGTATAATGCTAGCAGATGGTTAGTGTGCGTATCTGATACTTTTATGTCTGTCCTAC 100
                          >>>>>>>>>>>>>>>>>>>>>>>>>>>>>>>>>>>
                          J23119

           110       120       130       140       150       160       170       180       190       200
        *         *         *         *         *         *         *         *         *         *
101 AACTCCAAGCGTACAGTTCAGGCCAACACCCTAGCTTTGAGAAAGTCGCGTCAGTAAATGTCGTTTCCCTAAAGCTTACAATTTACCTCCTCGCTATTGG 200

           210       220       230       240       250       260       270       280       290       300
        *         *         *         *         *         *         *         *         *         *
201 GTTTGTACCGTACACCACTGAGACCGCCGTCGTCGACAAGCCGGCCGActagtaAAAATTAAGAGGTATATATTAatgtcagaattaattaaagaaaata 300
                                                          >>>>>>>>>>>>>>>>>>>>>
                                                          RBS (C-Dog)
                                                                               M  S  E  L  I  K  E  N  M
                                                                               >>>>>>>>>>>>>>>>>>>>>>>>>
                                                                               mKate2

           310       320       330       340       350       360       370       380       390       400
        *         *         *         *         *         *         *         *         *         *
301 tgcacatgaaattatatatggaaggtactgtcaacaatcatcatttcaaatgcacatccgaaggtgaaggtaaaccatatgaaggcacacaaacaatgcg 400
      H  M  K  L  Y  M  E  G  T  V  N  N  H  H  F  K  C  T  S  E  G  E  G  K  P  Y  E  G  T  Q  T  M  R
    >>>>>>>>>>>>>>>>>>>>>>>>>>>>>>>>>>>>>>>>>>>>>>>>>>>>>>>>>>>>>>>>>>>>>>>>>>>>>>>>>>>>>>>>>>>>>>>>>>>>
    mKate2

           410       420       430       440       450       460       470       480       490       500
        *         *         *         *         *         *         *         *         *         *
401 catcaaagcagttgaaggtggacccctgccctttgcgtttgacattctcgcaacgagctttatgtacgggtctaaaacttttatcaatcacacccaaggc 500
     I  K  A  V  E  G  G  P  L  P  F  A  F  D  I  L  A  T  S  F  M  Y  G  S  K  T  F  I  N  H  T  Q  G
    >>>>>>>>>>>>>>>>>>>>>>>>>>>>>>>>>>>>>>>>>>>>>>>>>>>>>>>>>>>>>>>>>>>>>>>>>>>>>>>>>>>>>>>>>>>>>>>>>>>>
    mKate2

           510       520       530       540       550       560       570       580       590       600
        *         *         *         *         *         *         *         *         *         *
501 attcctgactttttttaaacagtcctttcctgaaggctttacctgggaacgtgtaacaacttatgaagatggcggtgtacttacagcaactcaagatacga 600
    I  P  D  F  F  K  Q  S  F  P  E  G  F  T  W  E  R  V  T  T  Y  E  D  G  G  V  L  T  A  T  Q  D  T  S
    >>>>>>>>>>>>>>>>>>>>>>>>>>>>>>>>>>>>>>>>>>>>>>>>>>>>>>>>>>>>>>>>>>>>>>>>>>>>>>>>>>>>>>>>>>>>>>>>>>>>
    mKate2

           610       620       630       640       650       660       670       680       690       700
        *         *         *         *         *         *         *         *         *         *
601 gtttacaagatggctgtctgatttacaatgttaaaatccgtggcgtaaatttcccgagtaacggacccgtaatgcaaaaaaaaactcttggttgggaagc 700
      L  Q  D  G  C  L  I  Y  N  V  K  I  R  G  V  N  F  P  S  N  G  P  V  M  Q  K  K  T  L  G  W  E  A
    >>>>>>>>>>>>>>>>>>>>>>>>>>>>>>>>>>>>>>>>>>>>>>>>>>>>>>>>>>>>>>>>>>>>>>>>>>>>>>>>>>>>>>>>>>>>>>>>>>>>
    mKate2

           710       720       730       740       750       760       770       780       790       800
        *         *         *         *         *         *         *         *         *         *
701 atcaacagaaaccttatatcctgcggacggtggcttagaaggacgcgcagacatggcactgaaattagttggaggcggtcatttaatctgcaacctgaaa 800
     S  T  E  T  L  Y  P  A  D  G  G  L  E  G  R  A  D  M  A  L  K  L  V  G  G  G  H  L  I  C  N  L  K
    >>>>>>>>>>>>>>>>>>>>>>>>>>>>>>>>>>>>>>>>>>>>>>>>>>>>>>>>>>>>>>>>>>>>>>>>>>>>>>>>>>>>>>>>>>>>>>>>>>>>
    mKate2

           810       820       830       840       850       860       870       880       890       900
        *         *         *         *         *         *         *         *         *         *
801 acaacctatcgttccaaaaaacccgctaaaaaccttaaaatgcctggagtatactatgttgatcgtcgcttagaacgtattaaagaagctgataaagaaa 900
    T  T  Y  R  S  K  K  P  A  K  N  L  K  M  P  G  V  Y  Y  V  D  R  R  L  E  R  I  K  E  A  D  K  E  T
    >>>>>>>>>>>>>>>>>>>>>>>>>>>>>>>>>>>>>>>>>>>>>>>>>>>>>>>>>>>>>>>>>>>>>>>>>>>>>>>>>>>>>>>>>>>>>>>>>>>>
    mKate2

           910       920       930       940       950       960       970       980       990      1000
        *         *         *         *         *         *         *         *         *         *
```

```
 901 cctacgttgaacaacatgaagtagccgtagcccgttattgtgaccttccgtcgaaattaggacatcgttgataactctagaagcggccgcgaattcgagt 1000
       Y  V  E  Q  H  E  V  A  V  A  R  Y  C  D  L  P  S  K  L  G  H  R  *  *
     >>>>>>>>>>>>>>>>>>>>>>>>>>>>>>>>>>>>>>>>>>>>>>>>>>>>>>>>>>>>>>>>>>>>>>>>>>>
     mKate2

            1010      1020      1030      1040      1050      1060      1070      1080      1090      1100
               *         *         *         *         *         *         *         *         *         *
1001 cactaagggctaactaactaattacgtagcaatcaactcactggctcaccttcacgggtgggcctttcttcggcacgggcaaattgctgaatattccttt 1100

            1110      1120      1130      1140      1150      1160      1170      1180      1190      1200
               *         *         *         *         *         *         *         *         *         *
1101 tcttagacgtcaggtggcatactagtagcggccgctgcaggagtcactaagggttagttagttagattagcagaaagtcaaaagcctccgaccggaggct 1200

            1210      1220      1230      1240      1250      1260      1270      1280      1290      1300
               *         *         *         *         *         *         *         *         *         *
1201 tttgactaaaacttcccttggggttatcattggggctcactcaaaggcggtaatcagataaaaaaaatccttagctttcgctaaggatgatttctgctag 1300

            1310      1320      1330      1340      1350      1360      1370      1380      1390      1400
               *         *         *         *         *         *         *         *         *         *
1301 agctgtcagaccaagtttacgagctcgcttggactcctgttgatagatccagtaatgacctcagaactccatctggatttgttcagaacgctcggttgcc 1400

            1410      1420      1430      1440      1450      1460      1470      1480      1490      1500
               *         *         *         *         *         *         *         *         *         *
1401 gccgggcgttttttattggtgagaatccaagcactagggacagtaagacgggtaagcctgttgatgataccgctgccttactgggtgcattagccagtct 1500

            1510      1520      1530      1540      1550      1560      1570      1580      1590      1600
               *         *         *         *         *         *         *         *         *         *
1501 gaatgacctgtcacgggataatccgaagtggtcagactggaaaatcagagggcaggaactgctgaacagcaaaaagtcagatagcaccacatagcagacc 1600

            1610      1620      1630      1640      1650      1660      1670      1680      1690      1700
               *         *         *         *         *         *         *         *         *         *
1601 cgccataaaacgccctgagaagcccgtgacgggcttttcttgtattatgggtagtttccttgcatgaatccataaaaggcgcctgtagtgccatttaccc 1700

            1710      1720      1730      1740      1750      1760      1770      1780      1790      1800
               *         *         *         *         *         *         *         *         *         *
1701 ccattcactgccagagccgtgagcgcagcgaactgaatgtcacgaaaaagacagcgactcaggtgcctgatggtcggagacaaaaggaatattcagcgat 1800

            1810      1820      1830      1840      1850      1860      1870      1880      1890      1900
               *         *         *         *         *         *         *         *         *         *
1801 ttgcccgagcttgcgagggtgctacttaagcctttagggttttaaggtctgttttgtagaggagcaaacagcgtttgcgacatccttttgtaatactgcg 1900

            1910      1920      1930      1940      1950      1960      1970      1980      1990      2000
               *         *         *         *         *         *         *         *         *         *
1901 gaactgactaaagtagtgagttatacacagggctgggatctattctttttatctttttttattctttctttattctataaattataaccacttgaatata 2000

            2010      2020      2030      2040      2050      2060      2070      2080      2090      2100
               *         *         *         *         *         *         *         *         *         *
2001 aacaaaaaaaacacacaaaggtctagcggaatttacagagggtctagcagaatttacaagttttccagcaaaggtctagcagaatttacagatacccaca 2100

            2110      2120      2130      2140      2150      2160      2170      2180      2190      2200
               *         *         *         *         *         *         *         *         *         *
2101 actcaaaggaaaaggacatgtaattatcattgactagcccatctcaattggtatagtgattaaaatcacctagaccaattgagatgtatgtctgaattag 2200

            2210      2220      2230      2240      2250      2260      2270      2280      2290      2300
               *         *         *         *         *         *         *         *         *         *
2201 ttgttttcaaagcaaatgaactagcgattagtcgctatgacttaacggagcatgaaaccaagctaattttatgctgtgtggcactactcaaccccacgat 2300

            2310      2320      2330      2340      2350      2360      2370      2380      2390      2400
               *         *         *         *         *         *         *         *         *         *
2301 tgaaaaccctacaaggaaagaacggacggtatcgttcacttataaccaatacgctcagatgatgaacatcagtagggaaaatgcttatggtgtattagct 2400

            2410      2420      2430      2440      2450      2460      2470      2480      2490      2500
               *         *         *         *         *         *         *         *         *         *
2401 aaagcaaccagagagctgatgacgagaactgtggaaatcaggaatcctttggttaaaggctttgagattttccagtggacaaactatgccaagttctcaa 2500

            2510      2520      2530      2540      2550      2560      2570      2580      2590      2600
```

```
              *         *         *         *         *         *         *         *         *         *
2501 gcgaaaaattagaattagtttttagtgaagagatattgccttatcttttccagttaaaaaaattcataaaatataatctggaacatgttaagtcttttga 2600

                 2610      2620      2630      2640      2650      2660      2670      2680      2690      2700
              *         *         *         *         *         *         *         *         *         *
2601 aaacaaatactctatgaggatttatgagtggttattaaaagaactaacacaaaagaaaactcacaaggcaaatatagagattagccttgatgaatttaag 2700

                 2710      2720      2730      2740      2750      2760      2770      2780      2790      2800
              *         *         *         *         *         *         *         *         *         *
2701 ttcatgttaatgcttgaaaataactaccatgagtttaaaaggcttaaccaatgggttttgaaaccaataagtaaagatttaaacacttacagcaatatga 2800

                 2810      2820      2830      2840      2850      2860      2870      2880      2890      2900
              *         *         *         *         *         *         *         *         *         *
2801 aattggtggttgataagcgaggccgcccgactgatacgttgattttccaagttgaactagatagacaaatggatctcgtaaccgaacttgagaacaacca 2900

                 2910      2920      2930      2940      2950      2960      2970      2980      2990      3000
              *         *         *         *         *         *         *         *         *         *
2901 gataaaaatgaatggtgacaaaataccaacaaccattacatcagattcctacctacgtaacggactaagaaaaacactacacgatgctttaactgcaaaa 3000

                 3010      3020      3030      3040      3050      3060      3070      3080      3090      3100
              *         *         *         *         *         *         *         *         *         *
3001 attcagctcaccagttttgaggcaaaatttttgagtgacatgcaaagtaagcatgatctcaatggttcgttctcatggctcacgcaaaaacaacgaacca 3100

                 3110      3120      3130      3140      3150      3160      3170      3180      3190      3200
              *         *         *         *         *         *         *         *         *         *
3101 cactagagaacatactggctaaatacggaaggatctgaggttcttatggctcttgtatctatcagtgaagcatcaagactaacaaacaaaagtagaacaa 3200

                 3210      3220      3230      3240      3250      3260      3270      3280      3290      3300
              *         *         *         *         *         *         *         *         *         *
3201 ctgttcaccgttagatatcaaagggaaaactgtccatatgcacagatgaaaacggtgtaaaaaagatagatacatcagagcttttacgagttttggtgc 3300

                 3310      3320      3330      3340      3350      3360      3370      3380      3390      3400
              *         *         *         *         *         *         *         *         *         *
3301 atttaaagctgttcaccatgaacagatcgacaatgtaactactagaggttgatcgggcacgtaagaggttccaactttcaccataatgaaataagatcac 3400

                 3410      3420      3430      3440      3450      3460      3470      3480      3490      3500
              *         *         *         *         *         *         *         *         *         *
3401 taccgggcgtatttttgagttatcgagattttcaggagctaaggaagctaaaatggagaaaaaaatcacgggatataccaccgttgatatatcccaatg 3500

                 3510      3520      3530      3540      3550      3560      3570      3580      3590      3600
              *         *         *         *         *         *         *         *         *         *
3501 gcatcgtaaagaacattttgaggcatttcagtcagttgctcaatgtacctataaccagaccgttcagctggatattacggcctttttaaagaccgtaaag 3600

                 3610      3620      3630      3640      3650      3660      3670      3680      3690      3700
              *         *         *         *         *         *         *         *         *         *
3601 aaaaataagcacaagttttatccggcctttattcacattcttgcccgcctgatgaacgctcacccggagtttcgtatggccatgaaagacggtgagctgg 3700

                 3710      3720      3730      3740      3750      3760      3770      3780      3790      3800
              *         *         *         *         *         *         *         *         *         *
3701 tgatctgggatagtgttcacccttgttacaccgttttccatgagcaaactgaaacgttttcgtccctctggagtgaataccacgacgatttccggcagtt 3800

                 3810      3820      3830      3840      3850      3860      3870      3880      3890      3900
              *         *         *         *         *         *         *         *         *         *
3801 tctccacatatattcgcaagatgtggcgtgttacggtgaaaacctggcctatttccctaaagggtttattgagaatatgtttttgtctcagccaatccc 3900

                 3910      3920      3930      3940      3950      3960      3970      3980      3990      4000
              *         *         *         *         *         *         *         *         *         *
3901 tgggtgagtttcaccagttttgatttaaacgtggccaatatggacaacttcttcgcccccgttttcacgatgggcaaatattatacgcaaggcgacaagg 4000

                 4010      4020      4030      4040      4050      4060      4070      4080      4090      4100
              *         *         *         *         *         *         *         *         *         *
4001 tgctgatgccgctggcgatccaggttcatcatgccgtttgtgatggcttccatgtcggccgcatgcttaatgaattacaacagtactgtgatgagtggca 4100

                 4110      4120      4130      4140      4150      4160      4170      4180      4190      4200
              *         *         *         *         *         *         *         *         *         *
4101 gggcggggcgtaataatactagctccggcaaaaaaacgggcaaggtgtcaccaccctgccctttttctttaaaaccgaaaagattacttcgcgtttgcca 4200
```

```
          4210      4220      4230      4240      4250      4260      4270      4280      4290      4300
       *         *         *         *         *         *         *         *         *         *
4201 cctgacgtctaagaaaaggaatattcagcaatttgcccgtgccgaagaaaggcccacccgtgaaggtgagccagtgagttgattgctacgtaattagtta 4300

          4310      4320      4330      4340
       *         *         *         *
4301 gttagcccttagtgactcgaattcgcggccgcttctagag 4340
```

Sun Aug 26, 2012 1:36 CEST
J23119-B0032-E0051-Litmus28i.ape
Text Map

```
             10        20        30        40        50        60        70        80        90       100
         *         *         *         *         *         *         *         *         *         *
   1 gttaactacgtcaggtggcacttttcggggaaatgtgcgcggaacccctatttgtttatttttctaaatacattcaaatatgtatccgctcatgagacaa 100

            110       120       130       140       150       160       170       180       190       200
         *         *         *         *         *         *         *         *         *         *
 101 taaccctgataaatgcttcaataatattgaaaaaggaagagtatgagtattcaacatttccgtgtcgcccttattcccttttttgcggcattttgccttc 200

            210       220       230       240       250       260       270       280       290       300
         *         *         *         *         *         *         *         *         *         *
 201 ctgtttttgctcacccagaaacgctggtgaaagtaaaagatgctgaagatcagttgggtgcacgagtgggttacatcgaactggatctcaacagcggtaa 300

            310       320       330       340       350       360       370       380       390       400
         *         *         *         *         *         *         *         *         *         *
 301 gatccttgagagttttcgccccgaagaacgttctccaatgatgagcacttttaaagttctgctatgtggcgcggtattatcccgtgttgacgccgggcaa 400
                                             M  S  T  F  K  V  L  L  C  G  A  V  L  S  R  V  D  A  G  Q
                                             >>>>>>>>>>>>>>>>>>>>>>>>>>>>>>>>>>>>>>>>>>>>>>>>>>>>>>>>>>>>
                                             AmpR

            410       420       430       440       450       460       470       480       490       500
         *         *         *         *         *         *         *         *         *         *
 401 gagcaactcggtcgccgcatacactattctcagaatgacttggttgagtactcaccagtcacagaaaagcatcttacggatggcatgacagtaagagaat 500
     E  Q  L  G  R  R  I  H  Y  S  Q  N  D  L  V  E  Y  S  P  V  T  E  K  H  L  T  D  G  M  T  V  R  E  L
     >>>>>>>>>>>>>>>>>>>>>>>>>>>>>>>>>>>>>>>>>>>>>>>>>>>>>>>>>>>>>>>>>>>>>>>>>>>>>>>>>>>>>>>>>>>>>>>>>
     AmpR

            510       520       530       540       550       560       570       580       590       600
         *         *         *         *         *         *         *         *         *         *
 501 tatgcagtgctgccataaccatgagtgataacactgcggccaacttacttctgacaacgatcggaggaccgaaggagctaaccgcttttttgcacaacat 600
       C  S  A  A  I  T  M  S  D  N  T  A  A  N  L  L  L  T  T  I  G  G  P  K  E  L  T  A  F  L  H  N  M
     >>>>>>>>>>>>>>>>>>>>>>>>>>>>>>>>>>>>>>>>>>>>>>>>>>>>>>>>>>>>>>>>>>>>>>>>>>>>>>>>>>>>>>>>>>>>>>>>>
     AmpR

            610       620       630       640       650       660       670       680       690       700
         *         *         *         *         *         *         *         *         *         *
 601 ggggatcatgtaactcgccttgatcgttgggaaccggagctgaatgaagccataccaaacgacgagcgtgacaccacgatgcctgtagcaatggcaaca 700
      G  D  H  V  T  R  L  D  R  W  E  P  E  L  N  E  A  I  P  N  D  E  R  D  T  T  M  P  V  A  M  A  T
     >>>>>>>>>>>>>>>>>>>>>>>>>>>>>>>>>>>>>>>>>>>>>>>>>>>>>>>>>>>>>>>>>>>>>>>>>>>>>>>>>>>>>>>>>>>>>>>>>
     AmpR

            710       720       730       740       750       760       770       780       790       800
         *         *         *         *         *         *         *         *         *         *
 701 acgttgcgcaaactattaactggcgaactacttactctagcttcccggcaacaattaatagactggatggaggcggataaagttgcaggaccacttctgc 800
     T  L  R  K  L  L  T  G  E  L  L  T  L  A  S  R  Q  Q  L  I  D  W  M  E  A  D  K  V  A  G  P  L  L  R
     >>>>>>>>>>>>>>>>>>>>>>>>>>>>>>>>>>>>>>>>>>>>>>>>>>>>>>>>>>>>>>>>>>>>>>>>>>>>>>>>>>>>>>>>>>>>>>>>>
     AmpR

            810       820       830       840       850       860       870       880       890       900
         *         *         *         *         *         *         *         *         *         *
 801 gctcggcccttccggctggctggtttattgctgataaatctggagccggtgagcgtgggtctcgcggtatcattgcagcactggggccagatggtaagcc 900
       S  A  L  P  A  G  W  F  I  A  D  K  S  G  A  G  E  R  G  S  R  G  I  I  A  A  L  G  P  D  G  K  P
     >>>>>>>>>>>>>>>>>>>>>>>>>>>>>>>>>>>>>>>>>>>>>>>>>>>>>>>>>>>>>>>>>>>>>>>>>>>>>>>>>>>>>>>>>>>>>>>>>
     AmpR

            910       920       930       940       950       960       970       980       990      1000
         *         *         *         *         *         *         *         *         *         *
 901 ctcccgtatcgtagttatctacacgacggggagtcaggcaactatggatgaacgaaatagacagatcgctgagataggtgcctcactgattaagcattgg 1000
      S  R  I  V  V  I  Y  T  T  G  S  Q  A  T  M  D  E  R  N  R  Q  I  A  E  I  G  A  S  L  I  K  H  W
     >>>>>>>>>>>>>>>>>>>>>>>>>>>>>>>>>>>>>>>>>>>>>>>>>>>>>>>>>>>>>>>>>>>>>>>>>>>>>>>>>>>>>>>>>>>>>>>>>
     AmpR

           1010      1020      1030      1040      1050      1060      1070      1080      1090      1100
         *         *         *         *         *         *         *         *         *         *
1001 taactgtcagaccaagtttactcatatatactttagattgatttaccccggttgataatcagaaaagccccaaaaacaggaagattgtataagcaaatat 1100

           1110      1120      1130      1140      1150      1160      1170      1180      1190      1200
```

```
         *         *         *         *         *         *         *         *         *         *
1101 ttaaattgtaaacgttaatattttgttaaaattcgcgttaaatttttgttaaatcagctcattttttaaccaataggccgaaatcggcaaaatcccttat 1200
       <<<<<<<<<<<<<<<<<<<<<<<<<<<<<<<<<<<<<<<<<<<<<<<<<<<<<<<<<<<<<<<<<<<<<<<<<<<<<<<<<<<<<<<<<<<<<<<<
       M13 origin

             1210      1220      1230      1240      1250      1260      1270      1280      1290      1300
         *         *         *         *         *         *         *         *         *         *
1201 aaatcaaaagaatagcccgagatagggttgagtgttgttccagtttggaacaagagtccactattaaagaacgtggactccaacgtcaaagggcgaaaaa 1300
     <<<<<<<<<<<<<<<<<<<<<<<<<<<<<<<<<<<<<<<<<<<<<<<<<<<<<<<<<<<<<<<<<<<<<<<<<<<<<<<<<<<<<<<<<<<<<<<<<<<<
     M13 origin

             1310      1320      1330      1340      1350      1360      1370      1380      1390      1400
         *         *         *         *         *         *         *         *         *         *
1301 ccgtctatcagggcgatggcccactacgtgaaccatcacccaaatcaagttttttggggtcgaggtgccgtaaagcactaaatcggaaccctaaagggag 1400
     <<<<<<<<<<<<<<<<<<<<<<<<<<<<<<<<<<<<<<<<<<<<<<<<<<<<<<<<<<<<<<<<<<<<<<<<<<<<<<<<<<<<<<<<<<<<<<<<<<<<
     M13 origin

             1410      1420      1430      1440      1450      1460      1470      1480      1490      1500
         *         *         *         *         *         *         *         *         *         *
1401 cccccgatttagagcttgacggggaaagcgaacgtggcgagaaaggaagggaagaaagcgaaaggagcgggcgctagggcgctggcaagtgtagcggtca 1500
     <<<<<<<<<<<<<<<<<<<<<<<<<<<<<<<<<<<<<<<<<<<<<<<<<<<<<<<<<<<<<<<<<<<<<<<<<<<<<<<<<<<<<<<<<<<<<<<<<<<<
     M13 origin

             1510      1520      1530      1540      1550      1560      1570      1580      1590      1600
         *         *         *         *         *         *         *         *         *         *
1501 cgctgcgcgtaaccaccacacccgccgcgcttaatgcgccgctacagggcgcgtaaaaggatctaggtgaagatcctttttgataatctcatgaccaaaa 1600
                                                                                             >>>>>>>>>>>>
                                                                                             ColE1 origin

     <<<<<<<<<<<<<<<<<<<<<<<<<<<<<<<<<<<<<<<<<<<<<<<<<<<<<<
     M13 origin

             1610      1620      1630      1640      1650      1660      1670      1680      1690      1700
         *         *         *         *         *         *         *         *         *         *
1601 tcccttaacgtgagttttcgttccactgagcgtcagaccccgtagaaaagatcaaaggatcttcttgagatcctttttttctgcgcgtaatctgctgctt 1700
     >>>>>>>>>>>>>>>>>>>>>>>>>>>>>>>>>>>>>>>>>>>>>>>>>>>>>>>>>>>>>>>>>>>>>>>>>>>>>>>>>>>>>>>>>>>>>>>>>>>>
     ColE1 origin

             1710      1720      1730      1740      1750      1760      1770      1780      1790      1800
         *         *         *         *         *         *         *         *         *         *
1701 gcaaacaaaaaaaccaccgctaccagcggtggtttgtttgccggatcaagagctaccaactctttttccgaaggtaactggcttcagcagagcgcagata 1800
     >>>>>>>>>>>>>>>>>>>>>>>>>>>>>>>>>>>>>>>>>>>>>>>>>>>>>>>>>>>>>>>>>>>>>>>>>>>>>>>>>>>>>>>>>>>>>>>>>>>>
     ColE1 origin

             1810      1820      1830      1840      1850      1860      1870      1880      1890      1900
         *         *         *         *         *         *         *         *         *         *
1801 ccaaatactgttcttctagtgtagccgtagttaggccaccacttcaagaactctgtagcaccgcctacatacctcgctctgctaatcctgttaccagtgg 1900
     >>>>>>>>>>>>>>>>>>>>>>>>>>>>>>>>>>>>>>>>>>>>>>>>>>>>>>>>>>>>>>>>>>>>>>>>>>>>>>>>>>>>>>>>>>>>>>>>>>>>
     ColE1 origin

             1910      1920      1930      1940      1950      1960      1970      1980      1990      2000
         *         *         *         *         *         *         *         *         *         *
1901 ctgctgccagtggcgataagtcgtgtcttaccgggttggactcaagacgatagttaccggataaggcgcagcggtcgggctgaacggggggttcgtgcac 2000
     >>>>>>>>>>>>>>>>>>>>>>>>>>>>>>>>>>>>>>>>>>>>>>>>>>>>>>>>>>>>>>>>>>>>>>>>>>>>>>>>>>>>>>>>>>>>>>>>>>>>
     ColE1 origin

             2010      2020      2030      2040      2050      2060      2070      2080      2090      2100
         *         *         *         *         *         *         *         *         *         *
2001 acagcccagcttggagcgaacgacctacaccgaactgagatacctacagcgtgagctatgagaaagcgccacgcttcccgaagggagaaaggcggacagg 2100
     >>>>>>>>>>>>>>>>>>>>>>>>>>>>>>>>>>>>>>>>>>>>>>>>>>>>>>>>>>>>>>>>>>>>>>>>>>>>>>>>>>>>>>>>>>>>>>>>>>>>
     ColE1 origin

             2110      2120      2130      2140      2150      2160      2170      2180      2190      2200
         *         *         *         *         *         *         *         *         *         *
2101 tatccggtaagcggcagggtcggaacaggagagcgcacgagggagcttccagggggaaacgcctggtatctttatagtcctgtcgggtttcgccacctct 2200
     >>>>>>>>>>>>>>>>>>>>>>>>>>>>>>>>>>>>>>>>>>>>>>>>>>>>>>>>>>>>>>>>>>>>>>>>>>>>>>>>>>>>>>>>>>>>>>>>>>>>
     ColE1 origin

             2210      2220      2230      2240      2250      2260      2270      2280      2290      2300
         *         *         *         *         *         *         *         *         *         *
```

```
2201 gacttgagcgtcgatttttgtgatgctcgtcaggggggcggagcctatggaaaaacgccagcaacgcggcctttttacggttcctggccttttgctggcc 2300
     >>>>>>>>>>>>>>>>>>>>>>>>>>>>>>>>>>>>>>>>>>>>>>>>>>>>>>>>>>>>>>>>>>>>>>>>>
     ColE1 origin

             2310      2320      2330      2340      2350      2360      2370      2380      2390      2400
         *         *         *         *         *         *         *         *         *         *
2301 ttttgctcacatgtaatgtgagttagctcactcattaggcaccccaggctttacactttatgcttccggctcgtatgttgtgtggaattgtgagcggata 2400

             2410      2420      2430      2440      2450      2460      2470      2480      2490      2500
         *         *         *         *         *         *         *         *         *         *
2401 acaatttcacacaggaaacagctatgaccatgattacgccaagctacgtaatacgactcactatagggcagatcttcgaatgcatcgcgcgcaccgtacg 2500

             2510      2520      2530      2540      2550      2560      2570      2580      2590      2600
         *         *         *         *         *         *         *         *         *         *
2501 tctcgaggaattcgcggccgcttctagagttgacagctagctcagtcctaggtataatgctagctagtagagtcacacaggaaagtactagatgaccatg 2600
                                                                                           M  T  M
                                                                                           >>>>>>>>>
                                                                                           E0051

                                 >>>>>>>>>>>>>>>>>>>>>>>>>>>>>>>>>>>>        >>>>>>>>>>>>>
                                 J23119                                      B0032

             2610      2620      2630      2640      2650      2660      2670      2680      2690      2700
         *         *         *         *         *         *         *         *         *         *
2601 attacggattcactggccgtcgttttacaacgtcgtgactgggaaaaccctggcgttacccaacttaatcgccttgcagcacatccccctttcgccagct 2700
     I  T  D  S  L  A  V  V  L  Q  R  R  D  W  E  N  P  G  V  T  Q  L  N  R  L  A  A  H  P  P  F  A  S  W
     >>>>>>>>>>>>>>>>>>>>>>>>>>>>>>>>>>>>>>>>>>>>>>>>>>>>>>>>>>>>>>>>>>>>>>>>>>>>>>>>>>>>>>>>>>>>>>>>>>>>
     E0051

             2710      2720      2730      2740      2750      2760      2770      2780      2790      2800
         *         *         *         *         *         *         *         *         *         *
2701 ggcgtaatagcgaagaggcccgcaccgatcgcccttcccaacagttgcgcagcctgaatggcgaatggcgcgcgggcggcagcgaaggcggcggcagcga 2800
       R  N  S  E  E  A  R  T  D  R  P  S  Q  Q  L  R  S  L  N  G  E  W  R  A  G  G  S  E  G  G  G  S  E
     >>>>>>>>>>>>>>>>>>>>>>>>>>>>>>>>>>>>>>>>>>>>>>>>>>>>>>>>>>>>>>>>>>>>>>>>>>>>>>>>>>>>>>>>>>>>>>>>>>>>
     E0051

             2810      2820      2830      2840      2850      2860      2870      2880      2890      2900
         *         *         *         *         *         *         *         *         *         *
2801 acatcatcatcatcatcatggcagcgaacgtaaaggagaagaacttttcactggagttgtcccaattcttgttgaattagatggtgatgttaatgggcac 2900
      H  H  H  H  H  H  G  S  E  R  K  G  E  E  L  F  T  G  V  V  P  I  L  V  E  L  D  G  D  V  N  G  H
     >>>>>>>>>>>>>>>>>>>>>>>>>>>>>>>>>>>>>>>>>>>>>>>>>>>>>>>>>>>>>>>>>>>>>>>>>>>>>>>>>>>>>>>>>>>>>>>>>>>>
     E0051

             2910      2920      2930      2940      2950      2960      2970      2980      2990      3000
         *         *         *         *         *         *         *         *         *         *
2901 aaattttctgtcagtggagagggtgaaggtgatgcaacatacggaaaacttacccttaaatttatttgcactactggaaaactacctgttccatggccaa 3000
     K  F  S  V  S  G  E  G  E  G  D  A  T  Y  G  K  L  T  L  K  F  I  C  T  T  G  K  L  P  V  P  W  P  T
     >>>>>>>>>>>>>>>>>>>>>>>>>>>>>>>>>>>>>>>>>>>>>>>>>>>>>>>>>>>>>>>>>>>>>>>>>>>>>>>>>>>>>>>>>>>>>>>>>>>>
     E0051

             3010      3020      3030      3040      3050      3060      3070      3080      3090      3100
         *         *         *         *         *         *         *         *         *         *
3001 cacttgtcactaccctgacctatggtgttcaatgctttgcgagatacccagatcatatgaaacagcatgactttttcaagagtgccatgcccgaaggtta 3100
       L  V  T  T  L  T  Y  G  V  Q  C  F  A  R  Y  P  D  H  M  K  Q  H  D  F  F  K  S  A  M  P  E  G  Y
     >>>>>>>>>>>>>>>>>>>>>>>>>>>>>>>>>>>>>>>>>>>>>>>>>>>>>>>>>>>>>>>>>>>>>>>>>>>>>>>>>>>>>>>>>>>>>>>>>>>>
     E0051

             3110      3120      3130      3140      3150      3160      3170      3180      3190      3200
         *         *         *         *         *         *         *         *         *         *
3101 tgtacaggaaagaactatatttttcaaagatgacgggaactacaagacacgtgctgaagtcaagtttgaaggtgatacccttgttaatagaatcgagtta 3200
      V  Q  E  R  T  I  F  F  K  D  D  G  N  Y  K  T  R  A  E  V  K  F  E  G  D  T  L  V  N  R  I  E  L
     >>>>>>>>>>>>>>>>>>>>>>>>>>>>>>>>>>>>>>>>>>>>>>>>>>>>>>>>>>>>>>>>>>>>>>>>>>>>>>>>>>>>>>>>>>>>>>>>>>>>
     E0051

             3210      3220      3230      3240      3250      3260      3270      3280      3290      3300
         *         *         *         *         *         *         *         *         *         *
3201 aaaggtattgattttaaagaagatggaaacattcttggacacaaattggaatacaactataactcacacaatgtatacatcatggcagacaaacaaaaga 3300
     K  G  I  D  F  K  E  D  G  N  I  L  G  H  K  L  E  Y  N  Y  N  S  H  N  V  Y  I  M  A  D  K  Q  K  N
     >>>>>>>>>>>>>>>>>>>>>>>>>>>>>>>>>>>>>>>>>>>>>>>>>>>>>>>>>>>>>>>>>>>>>>>>>>>>>>>>>>>>>>>>>>>>>>>>>>>>
     E0051
```

```
           3310      3320      3330      3340      3350      3360      3370      3380      3390      3400
         *         *         *         *         *         *         *         *         *         *
3301 atggaatcaaagttaacttcaaaattagacacaacattgaagatggaagcgttcaactagcagaccattatcaacaaaatactccaattggcgatggccc 3400
       G  I  K  V  N  F  K  I  R  H  N  I  E  D  G  S  V  Q  L  A  D  H  Y  Q  Q  N  T  P  I  G  D  G  P
     >>>>>>>>>>>>>>>>>>>>>>>>>>>>>>>>>>>>>>>>>>>>>>>>>>>>>>>>>>>>>>>>>>>>>>>>>>>>>>>>>>>>>>>>>>>>>>>>>>>
     E0051

           3410      3420      3430      3440      3450      3460      3470      3480      3490      3500
         *         *         *         *         *         *         *         *         *         *
3401 tgtccttttaccagacaaccattacctgtccacacaatctgccctttcgaaagatcccaacgaaaagagagaccacatggtccttcttgagtttgtaaca 3500
      V  L  L  P  D  N  H  Y  L  S  T  Q  S  A  L  S  K  D  P  N  E  K  R  D  H  M  V  L  L  E  F  V  T
     >>>>>>>>>>>>>>>>>>>>>>>>>>>>>>>>>>>>>>>>>>>>>>>>>>>>>>>>>>>>>>>>>>>>>>>>>>>>>>>>>>>>>>>>>>>>>>>>>>>
     E0051

           3510      3520      3530      3540      3550      3560      3570      3580      3590      3600
         *         *         *         *         *         *         *         *         *         *
3501 gctgctgggattacacatggcatggatgaactatacaaataataatactagtagcggccgctgcaggatatctggatccacgaagcttcccatggtgacg 3600
     A  A  G  I  T  H  G  M  D  E  L  Y  K  *  *
     >>>>>>>>>>>>>>>>>>>>>>>>>>>>>>>>>>>>>>>>>>>>>
     E0051

           3610      3620      3630      3640      3650      3660      3670      3680      3690      3700
         *         *         *         *         *         *         *         *         *         *
3601 tcaccggttctagatacctaggtgagctctggtaccctctagtcaaggcctatagtgagtcgtattacggactggccgtcgttttacaacgtcgtgactg 3700

           3710      3720      3730      3740      3750      3760      3770      3780      3790      3800
         *         *         *         *         *         *         *         *         *         *
3701 ggaaaaccctggcgttacccaacttaatcgccttgcagcacatccccctttcgccagctggcgtaatagcgaagaggcccgcaccgatcgcccttcccaa 3800

           3810      3820      3830      3840      3850      3860      3870
         *         *         *         *         *         *         *
3801 cagttgcgcagcctgaatggcgaatggcgcttcgcttggtaataaagcccgcttcggcgggctttttttt 3870
```
